# The effect of interviewer-respondent age difference on the reporting of sexual activity in the Demographic and Health Surveys: Analysis of data from 21 countries

**DOI:** 10.7189/jogh.13.04002

**Published:** 2023-01-20

**Authors:** Jeffrey W Rozelle, Mark J Meyer, Anne H McKenna, Hawa Obaje, John D Kraemer

**Affiliations:** 1 Spatial Sciences Institute, University of Southern California, Los Angeles, California, USA; 2 Georgetown University, Department of Mathematics and Statistics, Washington D.C., USA; 3 Independent Researcher, Oakland, California, USA; 4 Last Mile Health, Tubman Blvd., Monrovia, Liberia; 5 Georgetown University, Department of Health Management and Policy, Washington D.C., USA

## Abstract

**Background:**

Interviewer effects can have consequential impacts on survey data, particularly for reporting sensitive attitudes and behaviours such as sexual activity and drug use, yet these effects remain understudied in low- and middle-income countries. The Demographic and Health Surveys (DHS) present a unique opportunity to study interviewer effects on the self-report of sensitive topics in low- and middle-income countries by including interviewer characteristics data. This paper aims to narrow the gap in research on interviewer effects by studying the effects that age difference between interviewer and respondent and interviewer survey experience have on the reporting of ever having sexual intercourse.

**Methods:**

We used DHS data from 91 066 women and 56 336 men in 21 countries where the standard DHS was implemented among all women of reproductive age, and interviewer characteristics were included in the data set. Using a Bayesian cross-classified model with random intercepts for interviewer and cluster, we assessed whether the effect of an age difference of 10 years or greater was associated with a difference in self-report of ever having sexual intercourse, adjusting for respondent demographics.

**Results:**

There was a meaningful association between an age difference of greater than ten years and reporting of ever having had sexual intercourse in most countries for both genders after adjusting for interviewer age and experience, rural or urban cluster, and individual-level characteristics. Among women, the marginal posterior probability of reporting ever having sexual intercourse if the interviewer was ten years or more years older was lower for 17 of 19 countries (countries ranged from -12.50 to 3.90 percentage points). Among men, the marginal posterior probability was lower for 16 of 20 countries, ranging from -18.30 to 17.10 percentage points.

**Conclusions:**

In most countries, women and men were less likely to report ever having sexual activity if the interviewer was ten or more years older than them, adjusting for potential confounders. These findings have important implications for interpreting numerous sexual health indicators, such as unmet family planning needs and human immunodeficiency virus (HIV)/acquired immunodeficiency syndrome (AIDS) risk. Survey administrators may consider more careful interviewer-respondent characteristic matching or novel approaches like Audio Computer Assisted Self Interview to minimize interviewer-induced variance.

Interviewer effects on survey response is well-documented, particularly when seeking to elicit true responses around sensitive behaviour such as sexual activity or substance use [1-4]. Researchers widely acknowledge the difficulty and potential data quality problems in collecting survey data about socially undesirable attitudes and behaviour in the United States and Europe, yet critical examination of survey data from low- and middle-income countries (LMICs) has often been slower to materialize [5,6]. A growing body of research around survey methods (with a particular interest in Audio Computer Assisted Self Interview (ACASI)) suggests that there are opportunities for novel survey administration techniques [3,5,7-9]. Nevertheless, researchers, policy makers, and public health practitioners will likely rely heavily on interviewer collected survey data for the foreseeable future to develop and target programming, especially in LMICs. It is therefore vital to develop a deeper understanding of the impact of interviewer effects on response accuracy.

Interviewer-induced bias is more likely to occur for sensitive questions such as sexual behaviour and falls into role-restricted and role-independent [10,11]. Role-restricted interviewer effects refer to the introduction of bias as a result of conscious and unconscious interviewer conduct such as reacting to responses, probing sensitive questions differently within or between interviewers, or modifying questions to reduce workload [6,12]. Role-independent effects result from respondent bias toward interviewer’s social characteristics such as gender, ethnicity, age, or lack of trust in interviewer and must be observable by the respondent [10,13]. Even with training, participants often detect these characteristics and edit their responses as a result [1]. It can be difficult to discern whether interviewer effects are role-restricted or -independent, but interviewer’s age, education, race, gender, and more characteristics have been associated with non-response to questions about sexual behaviour [8].

The Demographic and Health Surveys Program (DHS) is one of the most widely cited data sets in LMICs. It is a gold standard data set for many population statistics, particularly where few other similar sources of data may be available. Since 2015, DHS data sets have included surveyor characteristics, which creates an opportunity to study interviewer effects at a global scale [14]. The DHS has published two reports on interviewer effects. Their 2018 report focused on data quality issues like non-response, time to completion, and other general indicators of quality [12]. A recent report extended this and found that more sensitive and complicated questions were associated with larger interviewer-induced variance when models considered the cross-classified structure of interviews within communities and interviewers [15].

There are few peer-reviewed studies of interviewer effects in the DHS. Leone et al. found that variance attributable to interviewers was usually larger than variance attributed to clusters [16], though they called for more research into the specific interviewer characteristics that caused this variance. Metheny and Stephenson found that most interviewer characteristics, except for interviewer experience, did not significantly affect reporting of intimate partner violence [17]. Each of these studies were important milestones in studying the DHS, and this paper seeks to extend these efforts still further as the first study to investigate specific interviewer characteristics across all DHS countries with available data.

Interviewer effects on the reporting of sexual activity from the DHS does not yet appear in the literature, despite calls for research into it [12]. Because pre-marital sexual activity is stigmatized in many contexts [18-20] it is especially vulnerable to interviewer-induced social desirability bias [21,22]. It is likely that some interviewer characteristics – and how they interact with respondent characteristics – may exacerbate the risk of bias. For example, there is robust evidence in higher income countries and some low income countries that responses about sexual activity can be modified by interviewer and respondent gender [23,24]. Age has also been long identified as a potential source of interviewer effects, though age difference is less thoroughly studied [24,25]. Accurate reporting of sexual behaviour is valuable both on its own, as an important public health indicator, and as a gateway question for other questions and indicators about contraception use and sexual health [26]. Bias in respondents reporting sexual activity may propagate to other items that are important for assessing other domains of sexual and reproductive health.

This study aims to determine associations between interviewer characteristics and respondents’ reporting of ever having sexual intercourse in the DHS, using data from 91 066 respondents to the women’s and 56 336 respondents to the men’s questionnaires across 21 countries.

## METHODS

### Data source

This study makes secondary use of data from 21 countries’ cross-sectional surveys that were administered through the DHS program. The DHS is a nationally representative household survey administered in low- and middle- income countries with publicly available data. Participants are typically selected using a two-stage cluster sampling design with probability of selection proportional to size of the cluster at the first stage [27]. The sampling and data collection approach is fully described on the DHS program web portal [28]. In all countries, respondents and interviewers are gender-matched: women are interviewed by female interviewers and men by male interviewers.

We used DHS Survey Search tool to identify all current and future standard surveys that included the interviewer characteristics data set. We then included all surveys that a) used the standard DHS questionnaire, b) collected data from all women of reproductive age, and c) included the requisite data to link interviewer characteristics to respondents. Additionally, we excluded data sets where fewer than three percent of never-married, never-union respondents reported ever having sexual activity because at DHS sample sizes, models failed to converge when reporting was exceptionally rare. Twenty-one DHS countries met the inclusion criteria (**Table 1**).

**Table 1:** Included surveys and descriptive statistics

Country (years)	Gender	Included, never-unioned respondents	Included interviewers	Mean interviewer age	Mean interviews per interviewer	Mean clusters per interviewer	% Interviewers with >3 clusters	% Reporting ever having intercourse
**Benin (2017-18)**	Women	3897	73	28.50	53.40	18.00	94.50	49.90
	Men	2906	37	29.90	78.50	18.00	94.60	53.90
**Burundi (2016-17)**	Women	6091	75	35.40	81.20	21.00	97.30	16.20
	Men	2825	31	37.40	91.10	19.00	96.80	27.10
**Cameroon (2018)**	Women	4915	68	29.20	72.30	21.00	98.50	58.10
	Men	3380	34	30.00	99.40	23.00	100.00	59.50
**Ethiopia (2016)**	Women	4250	131	24.70	32.40	13.00	100.00	13.10
	Men	4600	58	28.20	79.30	18.00	100.00	29.40
**The Gambia (2019-20)**	Women	3226	51	30.60	63.30	16.00	100.00	12.20
	Men	2384	22	32.10	108.40	15.00	95.50	46.50
**Guinea (2018)**	Women	2645	61	30.50	43.40	15.00	100.00	35.70
	Men	1761	39	34.60	45.20	14.00	94.90	50.40
**Haiti (2016-17)**	Women	5760	55	34.60	104.70	24.00	96.40	56.40
	Men	4608	30	35.00	153.60	29.00	100.00	75.30
**Liberia (2019-20)**	Women	2621	54	32.10	48.50	15.00	100.00	81.30
	Men	1543	29	34.40	53.20	14.00	96.60	64.70
**Mali (2018)**	Women	1816	71	30.20	25.60	10.00	95.80	34.40
	Men	1580	45	30.20	35.10	11.00	97.80	38.90
**Malawi (2015-16)**	Women	4993	140	24.10	35.70	16.00	99.30	44.10
	Men	2666	67	29.50	39.80	16.00	97.00	62.50
**Myanmar (2016)**	Women							
	Men	1693	50	26.80	33.90	20.00	96.00	12.20
**Nepal (2016)**	Women							
	Men	1341	19	28.20	70.60	20.00	94.70	30.30
**Nigeria (2018)**	Women	10 669	116	37.40	92.00	29.00	97.40	36.80
	Men	5105	74	35.50	69.00	28.00	100.00	31.20
**Philippines (2017)**	Women	8652	262	37.20	33.00	11.00	95.40	15.00
	Men							
**Rwanda (2019-20)**	Women	5961	56	34.20	106.40	25.00	96.40	33.30
	Men	2857	25	36.70	114.30	22.00	100.00	38.90
**Sierra Leone (2019)**	Women	4966	66	31.10	75.20	17.00	87.90	67.60
	Men	2906	50	31.40	58.10	19.00	98.00	62.70
**Timor-Leste (2016)**	Women	4412	75	27.30	58.80	19.00	100.00	3.80
	Men	1712	32	30.30	53.50	15.00	93.80	27.30
**Uganda (2016)**	Women	4567	86	29.70	53.10	24.00	98.80	44.10
	Men	2029	39	30.80	52.00	23.00	100.00	57.10
**South Africa (2016)**	Women	5134	91	33.00	56.40	18.00	100.00	79.90
	Men	2241	30	31.30	74.70	21.00	100.00	80.70
**Zambia (2018)**	Women	4105	66	38.00	62.20	21.00	100.00	58.90
	Men	4934	39	46.00	126.50	24.00	100.00	65.00
**Zimbabwe (2015)**	Women	2386	58	28.90	41.10	17.00	84.50	31.30
	Men	3265	60	30.00	54.40	17.00	81.70	49.40

All male and female respondents who had never been in a marriage or union with complete data were included. We excluded respondents who reported ever being married or in a union because essentially all such respondents reported sexual activity. Although inclusion criteria are never-married or in a union, we use “never-unioned” for ease of expression. Detailed reports of all respondents are available from the DHS Program [28].

### Outcome of interest

The outcome of interest is whether a never-unioned respondent has reported ever having sexual intercourse. This is measured dichotomously with a standard DHS question: “How old were you when you had sexual intercourse for the very first time?”, one response option of which is “Never had intercourse.”

### Explanatory variables

We examined all potential interviewer-level factors to identify those that were a) available across all surveys, b) had any variance between interviewers, and c) fit theory for factors that could influence reporting of having sexual intercourse. Ultimately, our final model included interviewer age (continuous), age difference between interviewer and respondent (dichotomized to interviewers being 10+ years older or not), and any previous DHS or other survey experience (dichotomous).

We hypothesized that the relationship between age difference and probability of reporting sensitive behaviour was not linear, and that there is likely a difference at which respondents would perceive an interviewer as older and be more inclined to edit their responses. We calculated the difference between interviewer age and respondent age, and coded the age difference as fewer than 10 years older and 10 or more years older. We also constructed a variable of five or more years older for sensitivity analyses.

The interviewer characteristics data set also includes information on previous DHS experience and “other survey experience” collecting data. We collapsed this into a single “any survey experience” variable.

We also adjusted for several potential confounders: respondent age (continuous), education level (categorized as no school, primary, secondary, more than secondary), wealth index in five quintiles, and residence type (dichotomous as rural or urban). These variables were included because there is a well-established link between these predictors and age at first sexual activity, and because of their potential to confound the relationship between the outcome and variables of interest [29].

### Analysis

We first performed Bayesian simple logistic regression analyses with ever reporting sexual intercourse as the dependent variable for men’s and women’s questionnaires in each country. Each model had one independent variable: either any previous survey experience (DHS or other), wealth index quintiles, rural, respondent age in years, respondent education level, interviewer age in years, interviewer age difference of 10 or more years, interviewer education level, and difference in native language between interviewer or respondent native language (Table S3 and Table S4 in the **Online Supplementary Document**).

Interviews are nested within interviewers and within sampling clusters. Thus, every cluster has multiple interviewers who also worked across multiple clusters. We therefore fit Bayesian multilevel, cross-classified logistic regression models to analyse the effects of interviewer characteristics on respondent level responses, as cross-classified models are conventional for this type of analysis [11,15]. Interviewer-specific intercept was included in the model along with cluster-specific intercepts. The inclusion of cluster- and interviewer-specific intercepts accounts for potential additional sources of variability induced by the sampling design, and controls for a probable scenario where interviewers are assigned to clusters with different latent propensities of reporting ever having sexual activity. Sampling weights were not used in the present analysis, as the research question does not pertain to a population estimate, but to the equally weighted interviewer-respondent interaction.

We also checked for interpenetration of interviewers across clusters to understand the potential for cluster level variance to confound the conclusions. Prior research suggests that three or more clusters per interviewer would yield sufficient interpenetration for modelling [30], and that interviewer effects often account for more homogenization than sampling or spatial clustering [31].

We let “y” be the probability that respondent “i” interviewed by interviewer “j” in cluster “k” reported ever having intercourse. The difference in the log odds of an interviewer-respondent age difference of more than 10 years is represented by β1, while log odds of interviewer level age and survey experience are represented by β5 and β6 respectively. Level one, respondent characteristics included are respondent age, wealth index, wealth index, and education level (β2, β3, β4) included as fixed effects. The cluster-specific fixed effect of residence type (urban or rural) is included as β7. Intercepts ςj and ςk are the cross-classified interviewer- and community-specific intercepts.

*logit* (*Pr*(*y_ijk_* = 1)) = β_0_ + β_1_ (*age diff*. >10 *yrs*) *_i_*_(_*_jk_*_)_ + β_2_ (*resp. age*) *_i_*_(_*_jk_*_)_ + β_3_ (*resp. edu*) *_i_*_(_*_jk_*_)_ + β_4_ (*wealth index*) *_i_*_(_*_jk_*_)_ + β_5_ (*int. age*) *_j_* + β_6_ (*svy.* exp) _j_ + β_7_ (*residence type*) *_k_* + *ς_j_* + *ς_k_*

Because there is little relevant published literature on our research question in this context, we used non-informative priors. Specifically, we placed an improper flat prior over the reals for population-level effects. For the interviewer- and cluster-specific intercepts, we placed mean zero normal priors with half Student-t3 priors on the standard deviations (the subscript on t3 denotes the degrees of freedom).

Models for each country and each respondent gender were fitted separately. Estimates for all models were produced using eight Markov Chain Monte Carlo chains, with 6000 warm-up iterations and 6000 retained posterior samples per chain resulting in 48 000 total posterior samples available for analysis. The Gelman-Rubin potential scale reduction factor was used to assess convergence across chains. All covariates across all included models had a potential scale reduction factor, sometimes referred to as R-hat, of 1.00 – demonstrating model convergence [32]. Two key sensitivity analyses were performed. First, we fitted the models using a half-Cauchy distribution for the interviewer- and cluster-specific intercept prior standard deviations, and results were similar (Table S7 and Table S8 in the **Online Supplementary Document**). Second, we changed the interviewer-respondent age difference to five years. Associations held in most countries, with the strength of the association somewhat muted, as expected (Table S9 and Table S10 in the **Online Supplementary Document**).

We summarize the results using the median of the posterior samples, the middle 95% of the posterior samples (95% credible interval (CrI)), and the posterior probability that the difference is greater than zero. Posterior probabilities close to either zero or one indicate statistical significance [32].

Because our models estimate odds ratios, we also used average marginal effects to convert observed odds ratios into differences in the probability respondents reported sexual activity for interviewers within 10 years and 10 or more years, adjusting for potential confounders held at their observed values. We did this to reduce the risk that effect sizes would be misinterpreted, which is common with odds ratios.

All Bayesian regression analyses were performed using the brms function in R 4.1.1, and the posterior package was used for summary statistics of the posterior distributions [33-35]. We used the brmsmargins command in the brmsmargins package to estimate average marginal effects [36,37].

## RESULTS

This analysis used data from 21 countries, 91 066 women, and 56 336 men (**Table 1**). There were more women (median per country (mdn) = 4567) than men (mdn = 2745.50) included for all countries except Ethiopia, Zambia and Zimbabwe where there were more men than women that met inclusion criteria. Correspondingly, there were more unique interviewers for women (mdn = 71 interviewers) than for men (mdn = 38 interviewers) in all countries except Zimbabwe. The mean (m) number of women’s interviews per interviewer ranged from 25.60 in Mali to 106.40 in Rwanda. For the men’s questionnaire, the mean number of interviews per interviewer ranged from 33.90 in Myanmar to 153.60 in Haiti.

Among those who were never married, there were also inter-country variations in reporting of ever having had sex. In most countries, more never-unioned men (mdn = 49.90%) than women (mdn = 36.80%) reported ever having intercourse. Among included countries, Timor-Leste had the lowest proportion of never-unioned female respondents who reported ever having sexual intercourse (3.80%), followed by the Philippines, with 15% of never-unioned respondents reporting ever being sexually active. Liberia had the highest proportion of never-unioned women reporting sexual activity (81.30%). The proportion of never-unioned male respondents who reported ever having intercourse ranged from 12.20% in Myanmar to 80.70% in South Africa. The majority of included respondents were younger than 25 across contexts (Table S1 and Table S2 in the **Online Supplementary Document**).

In the final model, several independent variables are associated with reporting ever having sex among never-unioned respondents in the countries we analysed (Table S5 and Table S6 in the **Online Supplementary Document** for full models). In all countries, respondent age was a critical predictor of reporting ever having sexual intercourse. Among the covariates that describe interviewer effects, an age difference of 10 or more years appears to generally maintain a consistent, negative trend (**Table 2** and **Table 3**).

**Table 2:** Posterior median adjusted odds for age difference of 10 years or less and interviewer and community level predictors of reporting ever having sexual intercourse among never-unioned women

Country (years)	Age difference >10 years		Interviewer years of age		Interviewer survey experience		Rural residency	
	aOR (95% CrI)*	P.P. >1†	aOR (95% CrI)*	P.P. >1†	aOR (95% CrI)*	P.P. >1†	aOR (95% CrI)*	P.P. >1†
Benin (2017-18)	0.66 (0.48-0.88)	0.003	1.00 (0.96-1.04)	0.472	1.13 (0.75-1.69)	0.715	1.05 (0.85-1.30)	0.672
Burundi (2016-17)	0.77 (0.59-1.00)	0.027	1.01 (0.98-1.03)	0.681	0.77 (0.45-1.35)	0.176	0.50 (0.39-0.64)	0
Cameroon (2018)	0.64 (0.47-0.87)	0.002	1.04 (0.99-1.11)	0.930	0.62 (0.33-1.16)	0.066	1.23 (0.92-1.64)	0.921
Ethiopia (2016)	0.61 (0.35-1.03)	0.032	1.01 (0.94-1.08)	0.596	1.05 (0.69-1.60)	0.588	0.41 (0.25-0.65)	<0.001
Gambia (2019-20)	0.76 (0.46-1.24)	0.138	1.00 (0.96-1.04)	0.504	1.03 (0.68-1.55)	0.55	0.49 (0.31-0.76)	<0.001
Guinea (2018)	0.76 (0.52-1.10)	0.071	1.03 (0.96-1.11)	0.817	1.12 (0.50-2.51)	0.609	0.86 (0.59-1.25)	0.214
Haiti (2017-18)	0.93 (0.75-1.14)	0.249	0.99 (0.97-1.00)	0.026	1.19 (0.87-1.65)	0.869	0.65 (0.53-0.80)	0
Liberia (2019-20)	0.55 (0.32-0.96)	0.018	0.99 (0.95-1.03)	0.278	0.65 (0.40-1.02)	0.029	1.06 (0.72-1.55)	0.611
Mali (2018)	0.93 (0.59-1.48)	0.379	1.02 (0.97-1.08)	0.778	1.35 (0.61-3.03)	0.769	0.95 (0.58-1.57)	0.430
Malawi (2015-16)	0.75 (0.52-1.08)	0.059	1.03 (0.99-1.06)	0.939	1.09 (0.82-1.45)	0.734	0.88 (0.72-1.08)	0.115
Nigeria (2018)	1.43 (1.11-1.84)	0.997	1.01 (0.95-1.06)	0.574	1.00 (0.35-2.81)	0.497	1.11 (0.94-1.30)	0.894
Philippines (2017)	0.91 (0.71-1.17)	0.227	0.98 (0.97-1.00)	0.007	0.93 (0.63-1.37)	0.352	0.60 (0.49-0.72)	0
Rwanda (2019-20)	0.78 (0.63-0.96)	0.010	1.01 (0.99-1.02)	0.888	0.97 (0.82-1.14)	0.337	0.67 (0.56-0.80)	0
Sierra Leone (2019)	0.72 (0.51-1.02)	0.031	0.98 (0.93-1.02)	0.149	0.81 (0.49-1.33)	0.197	1.08 (0.81-1.45)	0.700
Timor-Leste (2016)	0.25 (0.12-0.47)	0	1.09 (1.00-1.18)	0.978	0.66 (0.33-1.32)	0.118	0.83 (0.45-1.47)	0.264
Uganda (2016)	0.69 (0.55-0.87)	<0.001	1.00 (0.98-1.03)	0.5	0.79 (0.59-1.06)	0.06	1.08 (0.88-1.31)	0.778
South Africa (2016)	0.73 (0.53-1.00)	0.025	0.99 (0.96-1.01)	0.152	1.15 (0.77-1.74)	0.754	0.85 (0.65-1.10)	0.103
Zambia (2018)	1.15 (0.83-1.60)	0.807	0.99 (0.97-1.01)	0.21	0.96 (0.66-1.41)	0.424	1.10 (0.87-1.41)	0.790
Zimbabwe (2015)	0.40 (0.27-0.59)	0	1.08 (1.02-1.16)	0.992	0.66 (0.41-1.09)	0.05	0.95 (0.60-1.48)	0.409

aOR – adjusted odds ratio, CrI – credible intervals, P.P. – posterior probability

*Adjusted odds ratio (95% credible intervals). Odds ratios are estimated from the median of the posterior samples adjusted for other model predictors including interviewer respondent age difference of greater than 10 years, interviewer years of age, interviewer with previous survey experience, rural residency, respondent age, wealth index, and education level.

†Posterior probability that the odds ratio is greater than one. Constructed as the proportion of posterior samples where the odds are greater than 1. Posterior probabilities of exactly 0 or 1 are when no or all samples, respectively, were above 1.

**Table 3:** Posterior median adjusted odds for age difference of 10 years or less and interviewer and community level predictors of reporting ever having sexual intercourse among never-unioned men

Country (years)	Age difference >10 years		Interviewer years of age		Interviewer survey experience		Rural residency	
	aOR (95% CrI)*	P.P. >1†	aOR (95% CrI)*	P.P. >1†	aOR (95% CrI)*	P.P. >1†	aOR (95% CrI)*	P.P. >1†
Benin (2017-18)	0.52 (0.37-0.73)	<0.001	0.99 (0.92-1.06)	0.35	0.76 (0.35-1.65)	0.235	1.03 (0.79-1.35)	0.595
Burundi (2016-17)	1.04 (0.74-1.48)	0.594	1.01 (0.95-1.09)	0.668	1.14 (0.15-8.67)	0.552	0.63 (0.46-0.85)	<0.001
Cameroon (2018)	0.64 (0.46-0.90)	0.006	1.02 (0.92-1.13)	0.642	0.78 (0.28-2.16)	0.305	1.30 (0.98-1.72)	0.965
Ethiopia (2016)	0.43 (0.31-0.61)	0	1.02 (0.97-1.07)	0.811	0.56 (0.20-1.53)	0.123	0.68 (0.48-0.94)	0.011
Gambia (2019-20)	0.68 (0.49-0.94)	0.009	1.03 (0.99-1.07)	0.921	1.12 (0.66-1.96)	0.667	0.73 (0.52-1.01)	0.027
Guinea (2018)	1.48 (0.90-2.45)	0.938	1.02 (0.95-1.10)	0.722	0.66 (0.15-2.95)	0.295	1.02 (0.68-1.53)	0.541
Haiti (2017-18)	0.65 (0.49-0.86)	0.001	1.00 (0.98-1.02)	0.473	1.09 (0.76-1.57)	0.693	0.76 (0.59-0.96)	0.011
Liberia (2019-20)	0.80 (0.43-1.49)	0.246	1.00 (0.94-1.06)	0.509	0.95 (0.46-1.94)	0.444	0.72 (0.49-1.07)	0.051
Mali (2018)	0.60 (0.37-0.98)	0.02	1.03 (0.90-1.17)	0.662	1.64 (0.42-6.66)	0.765	0.67 (0.39-1.13)	0.066
Malawi (2015-16)	0.52 (0.35-0.76)	<0.001	1.01 (0.99-1.04)	0.797	0.92 (0.62-1.37)	0.347	0.96 (0.75-1.25)	0.387
Myanmar (2015-16)	0.29 (0.10-0.72)	0.003	1.04 (1.00-1.09)	0.968	0.79 (0.43-1.41)	0.209	0.70 (0.47-1.06)	0.045
Nepal (2016)	0.55 (0.34-0.89)	0.007	1.05 (0.97-1.14)	0.874	1.35 (0.26-6.71)	0.651	1.33 (0.92-1.94)	0.936
Nigeria (2018)	1.09 (0.78-1.53)	0.699	0.98 (0.9-1.07)	0.328	1.06 (0.28-4.09)	0.536	1.20 (0.94-1.51)	0.930
Rwanda (2019-20)	0.80 (0.57-1.11)	0.091	0.99 (0.97-1.02)	0.346	0.58 (0.35-0.96)	0.018	0.67 (0.53-0.85)	<0.001
Sierra Leone (2019)	0.93 (0.61-1.40)	0.361	1.01 (0.95-1.07)	0.65	1.08 (0.53-2.22)	0.587	1.13 (0.79-1.64)	0.754
Timor-Leste (2016)	0.28 (0.17-0.45)	0	1.06 (0.94-1.19)	0.847	1.20 (0.30-4.87)	0.606	0.46 (0.29-0.73)	<0.001
Uganda (2016)	0.47 (0.32-0.69)	<0.001	1.02 (0.98-1.06)	0.809	0.71 (0.39-1.29)	0.129	1.23 (0.90-1.66)	0.902
South Africa (2016)	0.22 (0.13-0.35)	0	1.06 (1.02-1.10)	0.995	1.07 (0.48-2.38)	0.565	0.84 (0.55-1.26)	0.194
Zambia (2018)	3.14 (1.61-5.88)	>0.999	0.97 (0.94-1.00)	0.036	0.44 (0.07-2.61)	0.181	1.52 (1.22-1.90)	>0.999
Zimbabwe (2015)	0.50 (0.38-0.64)	0	1.03 (1.00-1.06)	0.965	0.68 (0.49-0.93)	0.008	1.06 (0.79-1.43)	0.649

aOR – adjusted odds ratio, CrI – credible intervals, P.P. – posterior probability

*Adjusted odds ratio (95% credible intervals). Odds ratios are estimated from the median of the posterior samples adjusted for other model predictors including interviewer respondent age difference of greater than 10 years, interviewer years of age, interviewer with previous survey experience, rural residency, respondent age, wealth index, and education level.

†Posterior probability that the odds ratio is greater than one. Constructed as the proportion of posterior samples where the odds are greater than 1. Posterior probabilities of exactly 0 or 1 are when no or all samples, respectively, were above 1.

In most countries, both women (17 of 19 countries) and men (16 of 20 countries) are less likely to report ever having sexual intercourse if the interviewer is 10 or more years older than they are, adjusting for interviewer age, survey experience, and respondent age and other demographics. We consider a posterior probability of 5% or lower that the coefficient was less than 0 to suggest statistical significance, although this is not strictly the same as frequentist statistical significance [32]. For female respondents, having an interviewer who was 10 or more years older had a significantly negative effect on odds of reporting of ever having sexual activity in Benin, Burundi, Cameroon, Ethiopia, Liberia, Malawi, Rwanda, Sierra Leone, Timor-Leste, Uganda, South Africa, and Zimbabwe (12 out of 19 included countries). For men, the effect was significantly negative in Benin, Cameroon, Ethiopia, The Gambia, Haiti, Mali, Malawi, Myanmar, Nepal, Timor-Leste, Uganda, South Africa, and Zimbabwe (13 out of 20 countries). In most countries the posterior median for the adjusted odds ratio is lower for men than for women.

The influence of previous survey experience is less clear (**Table 2** and **Table 3**, and Figure S1 in the **Online Supplementary Document**). Similarly, rural residency and interviewer age in years do not exhibit a consistent association. While living in a rural community does seem to have more influence in some countries than other community and interviewer level variables, the direction of the association is inconsistent between countries. In seven of 19 women’s and nine of 20 men’s questionnaires, the medians of the posterior adjusted odds suggest a positive relationship between living in a rural community with reporting intercourse, all others are negative.

**Figure 1:**
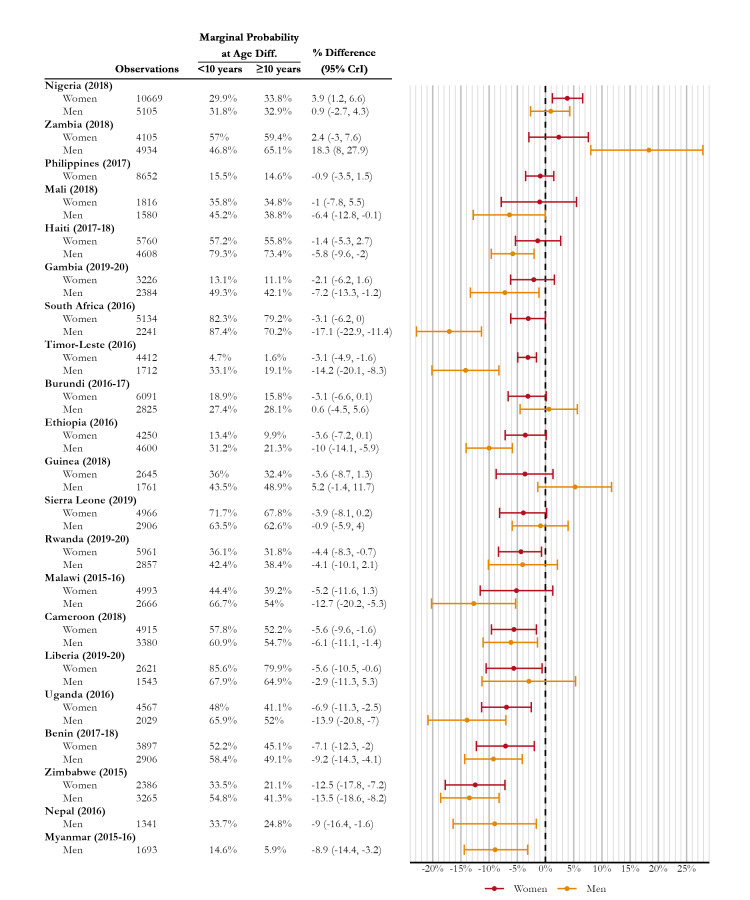
Average marginal effect contrasts of reporting ever having sexual intercourse among never-union respondents when an interviewer is 10 or more years older and fewer than 10 years, adjusting for respondent and interviewer characteristics.

**Figure 1** shows the difference in average marginal effect of having an interviewer that is 10 or more years older, adjusting for interviewer experience and age, respondent characteristics, and integrating out interviewer- and cluster-specific intercepts. The median of the posterior distribution of the average marginal effect contrasts among female respondents ranged from 3.90% (95% CrI = 1.20% to 6.60%) in Nigeria to -12.50% (95% CrI = -17.80% to -7.20%) in Zimbabwe. Excluding Zambia, an interviewer that is 10 or more years older than the respondent was associated with a difference in the probability of reporting sexual activity from 0.90% (95% CrI = -2.70% to 4.30%) probability in Nigeria to -17.10% (95% CrI = -22.90% to -11.40%) difference probability in South Africa.

The posterior medians of the difference in marginal effects were positive for Nigeria and Zambia. Given the vast differences in cultures and settings, this is not unexpected, and the 95% credible intervals for the male respondents in Zambia and female respondents in Zambia included zero.

One outstanding exception is for Zambia men, where the association is in the opposite direction and has a wide credible interval. The coefficient estimate for age difference of 10 or more years is substantially higher when including the random intercept for interviewers.

## DISCUSSION

To our knowledge, this is the first study to leverage the interviewer characteristics across multiple countries. Across countries and genders included in this analysis, respondents are less likely to report ever having had sex when an interviewer is 10 or more years older, controlling for respondent characteristics, interviewer age, experience, and cluster level variance. The other interviewer characteristic included in this analysis, previous survey experience, appears to have little association with reporting ever having sexual intercourse in most included countries.

Respondents in Nigeria, Zambia, and to the men’s questionnaire in Guinea were more likely to report ever having sexual activity to people more than 10 years older. Analyses with future DHS rounds in these and other included countries will shed light on the stability of the observed trends.

Response bias is well established when reporting sexual activity prior to a union in many contexts [5,8,38]. Where much of the extant literature has focused on non-response, scholars note that while cooperation is often more likely when interviewers and respondents are alike, cooperation does not necessarily imply candor [2,5,12]. Our findings align with literature from higher income countries that older interviewers elicit more conservative responses to sexual behaviour questions [24]. Studies have found inconsistencies from even the same respondent across self-reports, with differences that vary in directionality and magnitude by gender across contexts [39,40].

Previous research has also identified differences between reporting behaviour of men and women. Upchurch et al. found that both men and women can be inconsistent in their reporting of ever having sexual activity, but men were more likely to be inconsistent [41], whereas Soler-Hampejsek et al., found that women were more likely to be inconsistent in self-report [40]. Mensch et al. suggested that boys are more likely to exaggerate their sexual activity [5]. The results from the present analysis suggest a more nuanced perspective, that this bias is likely to be moderated by external factors including interviewer characteristics, particularly age difference between interviewer and respondent. These inconsistencies highlight the importance of better understanding interviewer characteristics in relationship to self-reported outcomes’ validity and reliability.

The biases we detected from interviewer effects will be transferred across other population metrics which use sexual activity as a numerator or denominator component. These include common indicators around use of family planning and human immunodeficiency virus (HIV)/acquired immunodeficiency syndrome (AIDS) prevention behaviour among young, or never married segments of the population. Although we did not examine other outcomes, it is plausible that interviewer effects are present in other sensitive outcomes and those who have inconsistent reporting on sexual behaviour are likely to have inconsistent reporting on other measures [4]. Metheny and Stephenson found that previous survey experience influenced reporting of intimate partner violence in Zimbabwe [17], and others have detected interviewer effects in the reporting of abortion [16,42]. This bias may lead to inaccuracies in estimates of the need for interventions among the populations that have the most to gain from such programs.

Population estimates may be improved with protocols that are more sensitive to interviewer effects and with novel collection modes. Mitigation strategies might include adding age and other characteristics when matching interviewers to respondents where feasible. Survey administrators may also wish to review interviewer training considering how to minimize role-restricted bias. ACASI is another promising tactic to address both role-independent and role-restricted interviewer biases. ACASI has been shown to improve accuracy and reduce inter-interviewer variance on sensitive questions in rural and semi-literate settings, though it may not be suitable in all contexts [5,8,43]. The DHS program may examine how to integrate these modalities in a manner that is accessible across populations.

There are salient limitations to this analysis. Although we were able to examine some key background effects, there was little information on mediators, such as interviewer attitudes, beliefs, or behaviours. This analysis also does not determine what is true. For example, although underreporting is typically considered to be a greater threat to validity for questions about sexual behaviour [24], it is also plausible that respondents may exaggerate their sexual experience to someone who is within 10 years of age.

Finally, key data limitations must be acknowledged. Several data sets either did not include characteristics for some interviewers or had improperly entered interviewer IDs – although the proportion of observations in affected data sets was small. It is also possible that, at the country level, models could be improved with the inclusion of variables that we did not include. Finally, upon advice from the DHS team, we removed observations where interviewers were misclassified, and missingness of individual variables is described in the respondent characteristics.

Finally, we note that this research is exploratory in nature. While there is sufficient consistency across surveys that the risk of age discrepancy-induced response bias should be taken seriously, this research would benefit from replication across contexts. In particular, because age can be a proxy for other sociodemographic characteristics, it would be valuable to extend this analysis with surveys that have more detailed interviewer data than DHS is able to collect. We suspect that there may be additional contextual factors that may modify interviewer effects, and this is an area rich for future research.

Kianersi et al. noted that much of the scholarship on interview effects is now relatively old, while attitudes, sensitivity and norms are shifting and the public health community should maintain a contemporary understanding of interviewer effects on the reporting of sexual activity [23]. Future research into the effects of interviewer characteristics on responses to sensitive questions in LMICs should be ongoing and focus on improving collection methods, better understanding what interviewer characteristics make response editing more likely, and re-evaluating estimates of indicators that may be vulnerable to interviewer effects. The DHS program could facilitate more robust research into interviewer-induced bias by standardizing and expanding interviewer characteristics data sets and matching response categories with the survey questionnaires where relevant (i.e. interviewer native language and ethnicity). This practice would also support replication of these results and analysis of trends in these effects over time.

## CONCLUSIONS

In most countries, women and men are less likely to report ever having sex when an interviewer is 10 or more years older when controlling for respondent characteristics. This has meaningful implications on the interpretation of this indicator, but also makes a strong case that the impact of interviewer effects should be considered more broadly in the DHS and other similar surveys. DHS could improve our understanding of these issues by collecting existing interviewer characteristics more consistently and adding several more dimensions. Survey implementers may consider better matching or careful training, or the assistance of technology like ACASI to reduce interviewer effects. Analysts may consider including a random intercept for interviewers when using DHS and similar survey data.

## Additional material


Online Supplementary Document
