# Supplementary material for: The effect of interviewer-respondent age difference on the reporting of sexual activity in the Demographic and Health Surveys: Analysis of data from 21 countries
Source: J Glob Health. 2023 Jan 20;13:04002. doi: 10.7189/jogh.13.04002 (PMC9850865; doi:10.7189/jogh.13.04002)
Supplement: Online Supplementary Document [file jogh-13-04002-s001.pdf]

The effect of interviewer-respondent age difference on the reporting of sexual activity in the Demographic and Health Surveys: Analysis of data from 21 countries

Online Supplementary Document

Respondent Characteristics

Table S1.	Descriptive statistics of **women's** survey respondent characteristics by outcome: numerator (%).	Page 2
Table S2.	Descriptive statistics of **men's** survey respondent characteristics by outcome: numerator (%).	Page 7

Simple Logistic Regression Models

Table S3.	Odds ratios, 95% credible intervals, and posterior probability that the odds are greater than 1* – **Women's** survey	Page 11
Table S4.	Odds ratios, 95% credible intervals, and posterior probability that the odds are greater than 1* – **Men's** survey	Page 14

Full Models

Table S5.	Log odds, 95% credible intervals, and posterior probability that log odds are greater than 0 from full model of ever reporting sexual intercourse among never-unioned **women**	Page 17
Table S6.	Log odds, 95% credible intervals, and posterior probability that log odds are greater than 0 from full model of ever reporting sexual intercourse among never-unioned **men**	Page 21

Sensitivity analysis models using half-Cauchy priors

Table S7.	Table S7. Log odds, 95% credible intervals, and posterior probability that log odds are greater than 0 from sensitivity model of ever reporting sexual intercourse among never-unioned **women**, using half-Cauchy priors	Page 25
Table S8.	Log odds, 95% credible intervals, and posterior probability that log odds are greater than 0 from sensitivity model of ever reporting sexual intercourse among never-unioned **men**, using half-Cauchy priors	Page 29

Sensitivity analysis models using age difference of five or more years

Table S9.	Table S9. Log odds, 95% credible intervals, and posterior probability that log odds are greater than 0 from sensitivity model of ever reporting sexual intercourse among never-unioned **women**, using age difference of five or more years	Page 33
Table S10.	Log odds, 95% credible intervals, and posterior probability that log odds are greater than 0 from sensitivity model of ever reporting sexual intercourse among never-unioned **men**, using age difference of five or more years	Page 37
Figure S1.	Average Marginal Effect contrasts of reporting ever having sexual intercourse among never-union respondents when an interviewer has previous survey experience and does not have survey experience, adjusting for respondent and interviewer characteristics.	Page 41

Respondent characteristics

Table S1. Descriptive statistics of women's survey respondent characteristics by outcome: numerator (%).

	Benin (2017-18)		Burundi (2016-17)		Cameroon (2018)		Ethiopia (2016)		Gambia (2019-20)	
	RESA*=0 N=1953	RESA*=1 N=1944	RESA*=0 N=5104	RESA*=1 N=987	RESA*=0 N=2058	RESA*=1 N=2857	RESA*=0 N=3692	RESA*=1 N=558	RESA*=0 N=2832	RESA*=1 N=394
**Age Group**										
15-19	1733 (88.7%)	968 (49.8%)	3309 (64.8%)	306 (31%)	1783 (86.6%)	899 (31.5%)	2580 (69.9%)	120 (21.5%)	1935 (68.3%)	68 (17.3%)
20-24	192 (9.8%)	699 (36%)	1212 (23.7%)	368 (37.3%)	210 (10.2%)	916 (32.1%)	723 (19.6%)	178 (31.9%)	636 (22.5%)	144 (36.5%)
25-29	19 (1%)	183 (9.4%)	387 (7.6%)	178 (18%)	43 (2.1%)	492 (17.2%)	253 (6.9%)	119 (21.3%)	205 (7.2%)	100 (25.4%)
30-34	5 (0.3%)	39 (2%)	94 (1.8%)	52 (5.3%)	12 (0.6%)	234 (8.2%)	76 (2.1%)	53 (9.5%)	44 (1.6%)	43 (10.9%)
35-39	2 (0.1%)	20 (1%)	58 (1.1%)	35 (3.5%)	7 (0.3%)	131 (4.6%)	32 (0.9%)	54 (9.7%)	11 (0.4%)	18 (4.6%)
40-44	1 (0.1%)	16 (0.8%)	26 (0.5%)	25 (2.5%)	2 (0.1%)	83 (2.9%)	19 (0.5%)	22 (3.9%)	1 (0%)	16 (4.1%)
45-49	1 (0.1%)	19 (1%)	18 (0.4%)	23 (2.3%)	1 (0%)	43 (1.5%)	9 (0.2%)	12 (2.2%)	0 (0%)	5 (1.3%)
50-54					0 (0%)	27 (0.9%)				
55-59					0 (0%)	17 (0.6%)				
**Interviewer had survey exp.**										
**Rural**	995 (50.9%)	918 (47.2%)	3916 (76.7%)	608 (61.6%)	796 (38.7%)	954 (33.4%)	1988 (53.8%)	123 (22%)	938 (33.1%)	87 (22.1%)
**≥ 10 year age difference**	1157 (59.2%)	691 (35.5%)	4295 (84.1%)	621 (62.9%)	1402 (68.1%)	977 (34.2%)	641 (17.4%)	28 (5%)	1522 (53.7%)	114 (28.9%)
**Wealth Index**	1157 (59.2%)	691 (35.5%)	4295 (84.1%)	621 (62.9%)	1402 (68.1%)	977 (34.2%)	641 (17.4%)	28 (5%)	1522 (53.7%)	114 (28.9%)
1	270 (13.8%)	194 (10%)	591 (11.6%)	117 (11.9%)	264 (12.8%)	92 (3.2%)	569 (15.4%)	35 (6.3%)	554 (19.6%)	58 (14.7%)
2	347 (17.8%)	276 (14.2%)	871 (17.1%)	116 (11.8%)	303 (14.7%)	424 (14.8%)	350 (9.5%)	25 (4.5%)	466 (16.5%)	62 (15.7%)
3	322 (16.5%)	343 (17.6%)	1029 (20.2%)	144 (14.6%)	447 (21.7%)	663 (23.2%)	446 (12.1%)	24 (4.3%)	496 (17.5%)	74 (18.8%)
4	413 (21.1%)	453 (23.3%)	1053 (20.6%)	176 (17.8%)	471 (22.9%)	757 (26.5%)	522 (14.1%)	34 (6.1%)	606 (21.4%)	87 (22.1%)
5	601 (30.8%)	678 (34.9%)	1560 (30.6%)	434 (44%)	573 (27.8%)	921 (32.2%)	1805 (48.9%)	440 (78.9%)	710 (25.1%)	113 (28.7%)
**Respondent education level**										
None	440 (22.5%)	417 (21.5%)	500 (9.8%)	142 (14.4%)	163 (7.9%)	92 (3.2%)	483 (13.1%)	43 (7.7%)	343 (12.1%)	45 (11.4%)
Primary	532 (27.2%)	415 (21.3%)	1856 (36.4%)	341 (34.5%)	437 (21.2%)	477 (16.7%)	1817 (49.2%)	177 (31.7%)	397 (14%)	52 (13.2%)
Secondary	948 (48.5%)	953 (49%)	2654 (52%)	473 (47.9%)	1349 (65.5%)	1842 (64.5%)	991 (26.8%)	180 (32.3%)	1859 (65.6%)	254 (64.5%)
> Secondary	33 (1.7%)	159 (8.2%)	94 (1.8%)	31 (3.1%)	109 (5.3%)	446 (15.6%)	401 (10.9%)	158 (28.3%)	233 (8.2%)	43 (10.9%)

* RESA: Reported ever having sexual activity

Table S1. Descriptive statistics of women's survey respondent characteristics by outcome: numerator (%). (continued)

	Guinea (2018)		Haiti (2017-18)		Liberia (2019-20)		Mali (2018)		Malawi (2015-16)	
	RESA*=0 N=1700	RESA*=1 N=945	RESA*=0 N=2513	RESA*=1 N=3247	RESA*=0 N=490	RESA*=1 N=2131	RESA*=0 N=1192	RESA*=1 N=624	RESA*=0 N=2790	RESA*=1 N=2203
**Age Group**										
15-19	1430 (84.1%)	394 (41.7%)	1950 (77.6%)	1084 (33.4%)	466 (95.1%)	974 (45.7%)	985 (82.6%)	288 (46.2%)	2452 (87.9%)	1244 (56.5%)
20-24	215 (12.6%)	289 (30.6%)	379 (15.1%)	1244 (38.3%)	21 (4.3%)	612 (28.7%)	150 (12.6%)	173 (27.7%)	287 (10.3%)	666 (30.2%)
25-29	34 (2%)	149 (15.8%)	109 (4.3%)	538 (16.6%)	2 (0.4%)	270 (12.7%)	40 (3.4%)	85 (13.6%)	33 (1.2%)	166 (7.5%)
30-34	16 (0.9%)	58 (6.1%)	45 (1.8%)	226 (7%)	1 (0.2%)	118 (5.5%)	11 (0.9%)	47 (7.5%)	9 (0.3%)	67 (3%)
35-39	5 (0.3%)	29 (3.1%)	15 (0.6%)	89 (2.7%)	0 (0%)	74 (3.5%)	3 (0.3%)	14 (2.2%)	4 (0.1%)	30 (1.4%)
40-44	0 (0%)	15 (1.6%)	9 (0.4%)	36 (1.1%)	0 (0%)	53 (2.5%)	2 (0.2%)	9 (1.4%)	3 (0.1%)	19 (0.9%)
45-49	0 (0%)	11 (1.2%)	6 (0.2%)	30 (0.9%)	0 (0%)	30 (1.4%)	1 (0.1%)	8 (1.3%)	2 (0.1%)	11 (0.5%)
50-54										
55-59										
**Interviewer had survey exp.**	1171 (68.9%)	683 (72.3%)	2281 (90.8%)	2997 (92.3%)	398 (81.2%)	1635 (76.7%)	955 (80.1%)	547 (87.7%)	962 (34.5%)	827 (37.5%)
**Rural**	803 (47.2%)	344 (36.4%)	1537 (61.2%)	1600 (49.3%)	238 (48.6%)	1001 (47%)	605 (50.8%)	261 (41.8%)	2113 (75.7%)	1513 (68.7%)
**≥ 10 year age difference**	1296 (76.2%)	472 (49.9%)	2024 (80.5%)	1876 (57.8%)	422 (86.1%)	1067 (50.1%)	774 (64.9%)	304 (48.7%)	480 (17.2%)	293 (13.3%)
**Wealth Index**										
1	214 (12.6%)	88 (9.3%)	474 (18.9%)	380 (11.7%)	73 (14.9%)	413 (19.4%)	213 (17.9%)	58 (9.3%)	349 (12.5%)	214 (9.7%)
2	249 (14.6%)	114 (12.1%)	539 (21.4%)	549 (16.9%)	98 (20%)	455 (21.4%)	161 (13.5%)	43 (6.9%)	399 (14.3%)	261 (11.8%)
3	250 (14.7%)	127 (13.4%)	483 (19.2%)	617 (19%)	125 (25.5%)	453 (21.3%)	143 (12%)	71 (11.4%)	501 (18%)	333 (15.1%)
4	392 (23.1%)	251 (26.6%)	469 (18.7%)	830 (25.6%)	91 (18.6%)	434 (20.4%)	255 (21.4%)	153 (24.5%)	558 (20%)	517 (23.5%)
5	595 (35%)	365 (38.6%)	548 (21.8%)	871 (26.8%)	103 (21%)	376 (17.6%)	420 (35.2%)	299 (47.9%)	983 (35.2%)	878 (39.9%)
**Respondent education level**										
None	663 (39%)	385 (40.7%)	58 (2.3%)	69 (2.1%)	47 (9.6%)	303 (14.2%)	483 (40.5%)	184 (29.5%)	40 (1.4%)	51 (2.3%)
Primary	342 (20.1%)	133 (14.1%)	786 (31.3%)	566 (17.4%)	285 (58.2%)	692 (32.5%)	199 (16.7%)	101 (16.2%)	1671 (59.9%)	969 (44%)
Secondary	627 (36.9%)	323 (34.2%)	1562 (62.2%)	2216 (68.2%)	150 (30.6%)	1032 (48.4%)	466 (39.1%)	303 (48.6%)	1023 (36.7%)	1029 (46.7%)
> Secondary	68 (4%)	104 (11%)	107 (4.3%)	396 (12.2%)	8 (1.6%)	104 (4.9%)	44 (3.7%)	36 (5.8%)	56 (2%)	154 (7%)

* RESA: Reported ever having sexual activity

Table S1. Descriptive statistics of women's survey respondent characteristics by outcome: numerator (%). (continued)

	**Nigeria (2018)**		**Philippines (2017)**		**Rwanda (2019-20)**		**Sierra Leone (2019)**		**Timor-Leste (2016)**	
	RESA*=0 N=6746	RESA*=1 N=3923	RESA*=0 N=7358	RESA*=1 N=1294	RESA*=0 N=3977	RESA*=1 N=1984	RESA*=0 N=1607	RESA*=1 N=3359	RESA*=0 N=4245	RESA*=1 N=167
**Age Group**										
15-19	5468 (81.1%)	1089 (27.8%)	4497 (61.1%)	163 (12.6%)	2721 (68.4%)	455 (22.9%)	1511 (94%)	1440 (42.9%)	2677 (63.1%)	20 (12%)
20-24	960 (14.2%)	1369 (34.9%)	1665 (22.6%)	398 (30.8%)	883 (22.2%)	689 (34.7%)	87 (5.4%)	1079 (32.1%)	951 (22.4%)	59 (35.3%)
25-29	227 (3.4%)	771 (19.7%)	567 (7.7%)	295 (22.8%)	220 (5.5%)	401 (20.2%)	8 (0.5%)	463 (13.8%)	328 (7.7%)	30 (18%)
30-34	54 (0.8%)	344 (8.8%)	249 (3.4%)	144 (11.1%)	76 (1.9%)	203 (10.2%)	0 (0%)	168 (5%)	120 (2.8%)	16 (9.6%)
35-39	24 (0.4%)	190 (4.8%)	156 (2.1%)	119 (9.2%)	46 (1.2%)	105 (5.3%)	0 (0%)	107 (3.2%)	61 (1.4%)	13 (7.8%)
40-44	8 (0.1%)	85 (2.2%)	108 (1.5%)	85 (6.6%)	19 (0.5%)	73 (3.7%)	1 (0.1%)	54 (1.6%)	63 (1.5%)	14 (8.4%)
45-49	5 (0.1%)	75 (1.9%)	116 (1.6%)	90 (7%)	12 (0.3%)	58 (2.9%)	0 (0%)	48 (1.4%)	45 (1.1%)	15 (9%)
50-54										
55-59										
**Interviewer had survey exp.**	5707 (84.6%)	3315 (84.5%)	6587 (89.5%)	1133 (87.6%)	2258 (56.8%)	1110 (55.9%)	1303 (81.1%)	2621 (78%)	2069 (48.7%)	65 (38.9%)
**Rural**	3286 (48.7%)	1903 (48.5%)	4518 (61.4%)	638 (49.3%)	2961 (74.5%)	1316 (66.3%)	702 (43.7%)	1407 (41.9%)	2473 (58.3%)	104 (62.3%)
**≥ 10 year age difference**	6274 (93%)	2770 (70.6%)	5350 (72.7%)	571 (44.1%)	2968 (74.6%)	977 (49.2%)	1456 (90.6%)	1782 (53.1%)	1513 (35.6%)	16 (9.6%)
**Wealth Index**										
1	816 (12.1%)	244 (6.2%)	1322 (18%)	133 (10.3%)	543 (13.7%)	362 (18.2%)	194 (12.1%)	390 (11.6%)	558 (13.1%)	29 (17.4%)
2	1098 (16.3%)	524 (13.4%)	1484 (20.2%)	227 (17.5%)	720 (18.1%)	325 (16.4%)	203 (12.6%)	447 (13.3%)	701 (16.5%)	29 (17.4%)
3	1510 (22.4%)	873 (22.3%)	1422 (19.3%)	316 (24.4%)	749 (18.8%)	310 (15.6%)	305 (19%)	623 (18.5%)	808 (19%)	33 (19.8%)
4	1606 (23.8%)	1141 (29.1%)	1506 (20.5%)	292 (22.6%)	810 (20.4%)	357 (18%)	437 (27.2%)	980 (29.2%)	1105 (26%)	37 (22.2%)
5	1716 (25.4%)	1141 (29.1%)	1624 (22.1%)	326 (25.2%)	1155 (29%)	630 (31.8%)	468 (29.1%)	919 (27.4%)	1073 (25.3%)	39 (23.4%)
**Respondent education level**										
None	882 (13.1%)	188 (4.8%)	40 (0.5%)	5 (0.4%)	62 (1.6%)	99 (5%)	209 (13%)	543 (16.2%)	382 (9%)	34 (20.4%)
Primary	624 (9.2%)	311 (7.9%)	423 (5.7%)	97 (7.5%)	1767 (44.4%)	1088 (54.8%)	355 (22.1%)	354 (10.5%)	378 (8.9%)	22 (13.2%)
Secondary	4642 (68.8%)	2472 (63%)	4227 (57.4%)	482 (37.2%)	2002 (50.3%)	685 (34.5%)	1025 (63.8%)	2238 (66.6%)	3042 (71.7%)	79 (47.3%)
> Secondary	598 (8.9%)	952 (24.3%)	2668 (36.3%)	709 (54.8%)	146 (3.7%)	112 (5.6%)	18 (1.1%)	224 (6.7%)	443 (10.4%)	32 (19.2%)

* RESA: Reported ever having sexual activity

Table S1. Descriptive statistics of women's survey respondent characteristics by outcome: numerator (%). (continued)

	Nigeria (2018)		Philippines (2017)		Rwanda (2019-20)		Sierra Leone (2019)		Timor-Leste (2016)	
	RESA*=0 N=6746	RESA*=1 N=3923	RESA*=0 N=7358	RESA*=1 N=1294	RESA*=0 N=3977	RESA*=1 N=1984	RESA*=0 N=1607	RESA*=1 N=3359	RESA*=0 N=4245	RESA*=1 N=167
**Age Group**										
15-19	5468 (81.1%)	1089 (27.8%)	4497 (61.1%)	163 (12.6%)	2721 (68.4%)	455 (22.9%)	1511 (94%)	1440 (42.9%)	2677 (63.1%)	20 (12%)
20-24	960 (14.2%)	1369 (34.9%)	1665 (22.6%)	398 (30.8%)	883 (22.2%)	689 (34.7%)	87 (5.4%)	1079 (32.1%)	951 (22.4%)	59 (35.3%)
25-29	227 (3.4%)	771 (19.7%)	567 (7.7%)	295 (22.8%)	220 (5.5%)	401 (20.2%)	8 (0.5%)	463 (13.8%)	328 (7.7%)	30 (18%)
30-34	54 (0.8%)	344 (8.8%)	249 (3.4%)	144 (11.1%)	76 (1.9%)	203 (10.2%)	0 (0%)	168 (5%)	120 (2.8%)	16 (9.6%)
35-39	24 (0.4%)	190 (4.8%)	156 (2.1%)	119 (9.2%)	46 (1.2%)	105 (5.3%)	0 (0%)	107 (3.2%)	61 (1.4%)	13 (7.8%)
40-44	8 (0.1%)	85 (2.2%)	108 (1.5%)	85 (6.6%)	19 (0.5%)	73 (3.7%)	1 (0.1%)	54 (1.6%)	63 (1.5%)	14 (8.4%)
45-49	5 (0.1%)	75 (1.9%)	116 (1.6%)	90 (7%)	12 (0.3%)	58 (2.9%)	0 (0%)	48 (1.4%)	45 (1.1%)	15 (9%)
50-54										
55-59										
**Interviewer had survey exp.**	5707 (84.6%)	3315 (84.5%)	6587 (89.5%)	1133 (87.6%)	2258 (56.8%)	1110 (55.9%)	1303 (81.1%)	2621 (78%)	2069 (48.7%)	65 (38.9%)
**Rural**	3286 (48.7%)	1903 (48.5%)	4518 (61.4%)	638 (49.3%)	2961 (74.5%)	1316 (66.3%)	702 (43.7%)	1407 (41.9%)	2473 (58.3%)	104 (62.3%)
**≥ 10 year age difference**	6274 (93%)	2770 (70.6%)	5350 (72.7%)	571 (44.1%)	2968 (74.6%)	977 (49.2%)	1456 (90.6%)	1782 (53.1%)	1513 (35.6%)	16 (9.6%)
**Wealth Index**										
1	816 (12.1%)	244 (6.2%)	1322 (18%)	133 (10.3%)	543 (13.7%)	362 (18.2%)	194 (12.1%)	390 (11.6%)	558 (13.1%)	29 (17.4%)
2	1098 (16.3%)	524 (13.4%)	1484 (20.2%)	227 (17.5%)	720 (18.1%)	325 (16.4%)	203 (12.6%)	447 (13.3%)	701 (16.5%)	29 (17.4%)
3	1510 (22.4%)	873 (22.3%)	1422 (19.3%)	316 (24.4%)	749 (18.8%)	310 (15.6%)	305 (19%)	623 (18.5%)	808 (19%)	33 (19.8%)
4	1606 (23.8%)	1141 (29.1%)	1506 (20.5%)	292 (22.6%)	810 (20.4%)	357 (18%)	437 (27.2%)	980 (29.2%)	1105 (26%)	37 (22.2%)
5	1716 (25.4%)	1141 (29.1%)	1624 (22.1%)	326 (25.2%)	1155 (29%)	630 (31.8%)	468 (29.1%)	919 (27.4%)	1073 (25.3%)	39 (23.4%)
**Respondent education level**										
None	882 (13.1%)	188 (4.8%)	40 (0.5%)	5 (0.4%)	62 (1.6%)	99 (5%)	209 (13%)	543 (16.2%)	382 (9%)	34 (20.4%)
Primary	624 (9.2%)	311 (7.9%)	423 (5.7%)	97 (7.5%)	1767 (44.4%)	1088 (54.8%)	355 (22.1%)	354 (10.5%)	378 (8.9%)	22 (13.2%)
Secondary	4642 (68.8%)	2472 (63%)	4227 (57.4%)	482 (37.2%)	2002 (50.3%)	685 (34.5%)	1025 (63.8%)	2238 (66.6%)	3042 (71.7%)	79 (47.3%)
> Secondary	598 (8.9%)	952 (24.3%)	2668 (36.3%)	709 (54.8%)	146 (3.7%)	112 (5.6%)	18 (1.1%)	224 (6.7%)	443 (10.4%)	32 (19.2%)

* RESA: Reported ever having sexual activity

Table S1. Descriptive statistics of women's survey respondent characteristics by outcome: numerator (%). (continued)

	**Uganda (2016)**		**South Africa (2016)**		**Zambia (2018)**		**Zimbabwe (2015)**	
	RESA*=0 N=2552	RESA*=1 N=2015	RESA*=0 N=1033	RESA*=1 N=4101	RESA*=0 N=1686	RESA*=1 N=2419	RESA*=0 N=1639	RESA*=1 N=747
**Age Group**								
15-19	2211 (86.6%)	950 (47.1%)	852 (82.5%)	609 (14.9%)	1443 (85.6%)	1028 (42.5%)	1312 (80%)	237 (31.7%)
20-24	270 (10.6%)	650 (32.3%)	124 (12%)	1036 (25.3%)	187 (11.1%)	828 (34.2%)	245 (14.9%)	254 (34%)
25-29	40 (1.6%)	247 (12.3%)	23 (2.2%)	865 (21.1%)	40 (2.4%)	347 (14.3%)	41 (2.5%)	136 (18.2%)
30-34	19 (0.7%)	73 (3.6%)	14 (1.4%)	567 (13.8%)	11 (0.7%)	119 (4.9%)	15 (0.9%)	65 (8.7%)
35-39	5 (0.2%)	47 (2.3%)	7 (0.7%)	416 (10.1%)	1 (0.1%)	48 (2%)	13 (0.8%)	21 (2.8%)
40-44	5 (0.2%)	32 (1.6%)	6 (0.6%)	342 (8.3%)	2 (0.1%)	32 (1.3%)	8 (0.5%)	14 (1.9%)
45-49	2 (0.1%)	16 (0.8%)	7 (0.7%)	266 (6.5%)	2 (0.1%)	17 (0.7%)	5 (0.3%)	20 (2.7%)
50-54								
55-59								
**Interviewer had survey exp.**	1816 (71.2%)	1348 (66.9%)	878 (85%)	3561 (86.8%)	849 (50.4%)	1241 (51.3%)	1022 (62.4%)	334 (44.7%)
**Rural**	1865 (73.1%)	1272 (63.1%)	455 (44%)	1941 (47.3%)	814 (48.3%)	1202 (49.7%)	847 (51.7%)	343 (45.9%)
**≥ 10 year age difference**	1735 (68%)	814 (40.4%)	853 (82.6%)	1399 (34.1%)	1493 (88.6%)	1715 (70.9%)	733 (44.7%)	124 (16.6%)
**Wealth Index**								
1	439 (17.2%)	243 (12.1%)	220 (21.3%)	911 (22.2%)	227 (13.5%)	353 (14.6%)	190 (11.6%)	83 (11.1%)
2	488 (19.1%)	264 (13.1%)	186 (18%)	981 (23.9%)	245 (14.5%)	400 (16.5%)	214 (13.1%)	86 (11.5%)
3	480 (18.8%)	319 (15.8%)	199 (19.3%)	981 (23.9%)	280 (16.6%)	440 (18.2%)	274 (16.7%)	109 (14.6%)
4	480 (18.8%)	415 (20.6%)	234 (22.7%)	818 (19.9%)	324 (19.2%)	524 (21.7%)	353 (21.5%)	200 (26.8%)
5	665 (26.1%)	774 (38.4%)	194 (18.8%)	410 (10%)	610 (36.2%)	702 (29%)	608 (37.1%)	269 (36%)
**Respondent education level**								
None	50 (2%)	42 (2.1%)	7 (0.7%)	62 (1.5%)	49 (2.9%)	69 (2.9%)	1 (0.1%)	7 (0.9%)
Primary	1574 (61.7%)	853 (42.3%)	79 (7.6%)	322 (7.9%)	609 (36.1%)	660 (27.3%)	202 (12.3%)	137 (18.3%)
Secondary	803 (31.5%)	770 (38.2%)	898 (86.9%)	3309 (80.7%)	972 (57.7%)	1484 (61.3%)	1345 (82.1%)	498 (66.7%)
> Secondary	125 (4.9%)	350 (17.4%)	49 (4.7%)	408 (9.9%)	56 (3.3%)	206 (8.5%)	91 (5.6%)	105 (14.1%)

* RESA: Reported ever having sexual activity

Table S2. Descriptive statistics of men's survey respondent characteristics by outcome: numerator (%).

	Benin (2017-18)		Burundi (2016-17)		Cameroon (2018)		Ethiopia (2016)		Gambia (2019-20)	
	RESA*=0	RESA*=1	RESA*=0	RESA*=1	RESA*=0	RESA*=1	RESA*=0	RESA*=1	RESA*=0	RESA*=1
	N=1340	N=1566	N=2059	N=766	N=1370	N=2010	N=3248	N=1352	N=1276	N=1108
**Age Group**										
15-19	1105 (82.5%)	442 (28.2%)	1236 (60%)	237 (30.9%)	1031 (75.3%)	475 (23.6%)	2000 (61.6%)	242 (17.9%)	818 (64.1%)	257 (23.2%)
20-24	192 (14.3%)	651 (41.6%)	577 (28%)	262 (34.2%)	232 (16.9%)	717 (35.7%)	848 (26.1%)	465 (34.4%)	302 (23.7%)	386 (34.8%)
25-29	32 (2.4%)	355 (22.7%)	172 (8.4%)	159 (20.8%)	82 (6%)	446 (22.2%)	292 (9%)	388 (28.7%)	112 (8.8%)	276 (24.9%)
30-34	7 (0.5%)	62 (4%)	57 (2.8%)	72 (9.4%)	16 (1.2%)	203 (10.1%)	75 (2.3%)	130 (9.6%)	31 (2.4%)	112 (10.1%)
35-39	2 (0.1%)	24 (1.5%)	5 (0.2%)	16 (2.1%)	3 (0.2%)	84 (4.2%)	19 (0.6%)	65 (4.8%)	11 (0.9%)	42 (3.8%)
40-44	1 (0.1%)	13 (0.8%)	6 (0.3%)	3 (0.4%)	2 (0.1%)	32 (1.6%)	6 (0.2%)	35 (2.6%)	2 (0.2%)	19 (1.7%)
45-49	1 (0.1%)	5 (0.3%)	2 (0.1%)	5 (0.7%)	3 (0.2%)	21 (1%)	3 (0.1%)	12 (0.9%)	0 (0%)	9 (0.8%)
50-54	0 (0%)	4 (0.3%)	1 (0%)	5 (0.7%)	1 (0.1%)	19 (0.9%)	2 (0.1%)	11 (0.8%)	0 (0%)	6 (0.5%)
55-59	0 (0%)	6 (0.4%)	3 (0.1%)	7 (0.9%)	0 (0%)	10 (0.5%)	3 (0.1%)	4 (0.3%)	0 (0%)	1 (0.1%)
**Interviewer had survey exp.**	930 (69.4%)	963 (61.5%)	1899 (92.2%)	696 (90.9%)	1050 (76.6%)	1439 (71.6%)	2993 (92.1%)	1202 (88.9%)	984 (77.1%)	869 (78.4%)
**Rural**	754 (56.3%)	780 (49.8%)	1536 (74.6%)	469 (61.2%)	665 (48.5%)	732 (36.4%)	2219 (68.3%)	539 (39.9%)	536 (42%)	327 (29.5%)
**≥ 10 year age difference**	815 (60.8%)	316 (20.2%)	1744 (84.7%)	494 (64.5%)	959 (70%)	681 (33.9%)	1196 (36.8%)	159 (11.8%)	677 (53.1%)	317 (28.6%)
**Wealth Index**										
1	231 (17.2%)	247 (15.8%)	204 (9.9%)	64 (8.4%)	261 (19.1%)	90 (4.5%)	665 (20.5%)	155 (11.5%)	315 (24.7%)	216 (19.5%)
2	298 (22.2%)	246 (15.7%)	299 (14.5%)	84 (11%)	278 (20.3%)	292 (14.5%)	422 (13%)	97 (7.2%)	280 (21.9%)	189 (17.1%)
3	265 (19.8%)	272 (17.4%)	354 (17.2%)	105 (13.7%)	343 (25%)	504 (25.1%)	487 (15%)	89 (6.6%)	237 (18.6%)	206 (18.6%)
4	254 (19%)	307 (19.6%)	456 (22.1%)	137 (17.9%)	245 (17.9%)	506 (25.2%)	549 (16.9%)	161 (11.9%)	211 (16.5%)	217 (19.6%)
5	292 (21.8%)	494 (31.5%)	746 (36.2%)	376 (49.1%)	243 (17.7%)	618 (30.7%)	1125 (34.6%)	850 (62.9%)	233 (18.3%)	280 (25.3%)
**Respondent education level**										
None	324 (24.2%)	335 (21.4%)	109 (5.3%)	68 (8.9%)	117 (8.5%)	60 (3%)	358 (11%)	84 (6.2%)	294 (23%)	187 (16.9%)
Primary	278 (20.7%)	279 (17.8%)	835 (40.6%)	301 (39.3%)	324 (23.6%)	342 (17%)	1772 (54.6%)	427 (31.6%)	260 (20.4%)	186 (16.8%)
Secondary	701 (52.3%)	758 (48.4%)	1051 (51%)	329 (43%)	874 (63.8%)	1275 (63.4%)	821 (25.3%)	421 (31.1%)	653 (51.2%)	621 (56%)
> Secondary	37 (2.8%)	194 (12.4%)	64 (3.1%)	68 (8.9%)	55 (4%)	333 (16.6%)	297 (9.1%)	420 (31.1%)	69 (5.4%)	114 (10.3%)

* RESA: Reported ever having sexual activity

Table S2. Descriptive statistics of men's survey respondent characteristics by outcome: numerator (%). (Continued)

	Guinea (2018)		Haiti (2017-18)		Liberia (2019-20)		Mali (2018)		Malawi (2015-16)	
	RESA*=0 N=874	RESA*=1 N=887	RESA*=0 N=1136	RESA*=1 N=3472	RESA*=0 N=544	RESA*=1 N=999	RESA*=0 N=966	RESA*=1 N=614	RESA*=0 N=1000	RESA*=1 N=1666
**Age Group**										
15-19	659 (75.4%)	292 (32.9%)	953 (83.9%)	1182 (34%)	492 (90.4%)	371 (37.1%)	709 (73.4%)	175 (28.5%)	826 (82.6%)	823 (49.4%)
20-24	142 (16.2%)	303 (34.2%)	124 (10.9%)	1223 (35.2%)	37 (6.8%)	378 (37.8%)	181 (18.7%)	237 (38.6%)	137 (13.7%)	634 (38.1%)
25-29	52 (5.9%)	177 (20%)	31 (2.7%)	578 (16.6%)	9 (1.7%)	138 (13.8%)	57 (5.9%)	138 (22.5%)	17 (1.7%)	140 (8.4%)
30-34	14 (1.6%)	73 (8.2%)	3 (0.3%)	269 (7.7%)	3 (0.6%)	66 (6.6%)	15 (1.6%)	36 (5.9%)	11 (1.1%)	42 (2.5%)
35-39	4 (0.5%)	27 (3%)	9 (0.8%)	91 (2.6%)	0 (0%)	19 (1.9%)	3 (0.3%)	13 (2.1%)	6 (0.6%)	11 (0.7%)
40-44	1 (0.1%)	7 (0.8%)	8 (0.7%)	51 (1.5%)	0 (0%)	15 (1.5%)	1 (0.1%)	9 (1.5%)	2 (0.2%)	6 (0.4%)
45-49	0 (0%)	4 (0.5%)	3 (0.3%)	34 (1%)	1 (0.2%)	8 (0.8%)	0 (0%)	3 (0.5%)	1 (0.1%)	7 (0.4%)
50-54	1 (0.1%)	2 (0.2%)	1 (0.1%)	19 (0.5%)	2 (0.4%)	3 (0.3%)	0 (0%)	0 (0%)	0 (0%)	3 (0.2%)
55-59	1 (0.1%)	2 (0.2%)	3 (0.3%)	17 (0.5%)	0 (0%)	1 (0.1%)	0 (0%)	3 (0.5%)		
**Interviewer had survey exp.**	757 (86.6%)	753 (84.9%)	844 (74.3%)	2590 (74.6%)	438 (80.5%)	762 (76.3%)	700 (72.5%)	463 (75.4%)	506 (50.6%)	772 (46.3%)
**Rural**	469 (53.7%)	381 (43%)	852 (75%)	2021 (58.2%)	334 (61.4%)	488 (48.8%)	621 (64.3%)	352 (57.3%)	771 (77.1%)	1242 (74.5%)
**≥ 10 year age difference**	683 (78.1%)	541 (61%)	870 (76.6%)	1700 (49%)	513 (94.3%)	676 (67.7%)	622 (64.4%)	219 (35.7%)	347 (34.7%)	350 (21%)
**Wealth Index**										
1	126 (14.4%)	86 (9.7%)	348 (30.6%)	529 (15.2%)	123 (22.6%)	158 (15.8%)	183 (18.9%)	66 (10.7%)	100 (10%)	192 (11.5%)
2	122 (14%)	106 (12%)	251 (22.1%)	665 (19.2%)	130 (23.9%)	229 (22.9%)	188 (19.5%)	91 (14.8%)	156 (15.6%)	243 (14.6%)
3	175 (20%)	141 (15.9%)	251 (22.1%)	774 (22.3%)	141 (25.9%)	255 (25.5%)	170 (17.6%)	97 (15.8%)	200 (20%)	306 (18.4%)
4	193 (22.1%)	226 (25.5%)	167 (14.7%)	714 (20.6%)	80 (14.7%)	182 (18.2%)	198 (20.5%)	160 (26.1%)	216 (21.6%)	370 (22.2%)
5	258 (29.5%)	328 (37%)	119 (10.5%)	790 (22.8%)	70 (12.9%)	175 (17.5%)	227 (23.5%)	200 (32.6%)	328 (32.8%)	555 (33.3%)
**Respondent education level**										
None	286 (32.7%)	256 (28.9%)	58 (5.1%)	125 (3.6%)	64 (11.8%)	83 (8.3%)	437 (45.2%)	175 (28.5%)	16 (1.6%)	32 (1.9%)
Primary	207 (23.7%)	121 (13.6%)	567 (49.9%)	793 (22.8%)	336 (61.8%)	263 (26.3%)	161 (16.7%)	89 (14.5%)	659 (65.9%)	880 (52.8%)
Secondary	351 (40.2%)	376 (42.4%)	504 (44.4%)	2214 (63.8%)	144 (26.5%)	590 (59.1%)	352 (36.4%)	288 (46.9%)	310 (31%)	689 (41.4%)
> Secondary	30 (3.4%)	134 (15.1%)	7 (0.6%)	340 (9.8%)	0 (0%)	63 (6.3%)	16 (1.7%)	62 (10.1%)	15 (1.5%)	65 (3.9%)

* RESA: Reported ever having sexual activity

Table S2. Descriptive statistics of men's survey respondent characteristics by outcome: numerator (%). (Continued)

	Myanmar (2015-16)		Nepal (2016)		Nigeria (2018)		Rwanda (2019-20)		Sierra Leone (2019)	
	RESA*=0	RESA*=1	RESA*=0	RESA*=1	RESA*=0	RESA*=1	RESA*=0	RESA*=1	RESA*=0	RESA*=1
	N=1487	N=206	N=935	N=406	N=3513	N=1592	N=1745	N=1112	N=1083	N=1823
**Age Group**										
15-19	712 (47.9%)	22 (10.7%)	718 (76.8%)	173 (42.6%)	2201 (62.7%)	263 (16.5%)	1231 (70.5%)	271 (24.4%)	985 (91%)	585 (32.1%)
20-24	389 (26.2%)	75 (36.4%)	172 (18.4%)	151 (37.2%)	815 (23.2%)	499 (31.3%)	383 (21.9%)	427 (38.4%)	81 (7.5%)	655 (35.9%)
25-29	171 (11.5%)	55 (26.7%)	35 (3.7%)	63 (15.5%)	339 (9.6%)	454 (28.5%)	94 (5.4%)	257 (23.1%)	13 (1.2%)	383 (21%)
30-34	97 (6.5%)	24 (11.7%)	6 (0.6%)	12 (3%)	108 (3.1%)	240 (15.1%)	24 (1.4%)	96 (8.6%)	1 (0.1%)	103 (5.7%)
35-39	54 (3.6%)	17 (8.3%)	3 (0.3%)	7 (1.7%)	29 (0.8%)	75 (4.7%)	5 (0.3%)	38 (3.4%)	1 (0.1%)	52 (2.9%)
40-44	43 (2.9%)	9 (4.4%)	1 (0.1%)	0 (0%)	10 (0.3%)	31 (1.9%)	2 (0.1%)	9 (0.8%)	0 (0%)	25 (1.4%)
45-49	21 (1.4%)	4 (1.9%)	0 (0%)	0 (0%)	8 (0.2%)	18 (1.1%)	1 (0.1%)	6 (0.5%)	1 (0.1%)	11 (0.6%)
50-54					1 (0%)	7 (0.4%)	3 (0.2%)	3 (0.3%)	1 (0.1%)	4 (0.2%)
55-59					2 (0.1%)	5 (0.3%)	2 (0.1%)	5 (0.4%)	0 (0%)	5 (0.3%)
**Interviewer had survey exp.**	753 (50.6%)	93 (45.1%)	886 (94.8%)	389 (95.8%)	2973 (84.6%)	1343 (84.4%)	1284 (73.6%)	697 (62.7%)	924 (85.3%)	1528 (83.8%)
**Rural**	1042 (70.1%)	118 (57.3%)	273 (29.2%)	141 (34.7%)	2088 (59.4%)	795 (49.9%)	1372 (78.6%)	722 (64.9%)	621 (57.3%)	846 (46.4%)
**≥ 10 year age difference**	254 (17.1%)	20 (9.7%)	484 (51.8%)	123 (30.3%)	2937 (83.6%)	891 (56%)	1436 (82.3%)	665 (59.8%)	867 (80.1%)	795 (43.6%)
**Wealth Index**										
1	225 (15.1%)	15 (7.3%)	156 (16.7%)	75 (18.5%)	794 (22.6%)	108 (6.8%)	237 (13.6%)	123 (11.1%)	185 (17.1%)	222 (12.2%)
2	257 (17.3%)	36 (17.5%)	176 (18.8%)	56 (13.8%)	724 (20.6%)	200 (12.6%)	332 (19%)	159 (14.3%)	198 (18.3%)	251 (13.8%)
3	360 (24.2%)	31 (15%)	170 (18.2%)	77 (19%)	760 (21.6%)	358 (22.5%)	377 (21.6%)	188 (16.9%)	234 (21.6%)	332 (18.2%)
4	335 (22.5%)	61 (29.6%)	223 (23.9%)	102 (25.1%)	707 (20.1%)	450 (28.3%)	393 (22.5%)	256 (23%)	244 (22.5%)	536 (29.4%)
5	310 (20.8%)	63 (30.6%)	210 (22.5%)	96 (23.6%)	528 (15%)	476 (29.9%)	406 (23.3%)	386 (34.7%)	222 (20.5%)	482 (26.4%)
**Respondent education level**										
None	112 (7.5%)	10 (4.9%)	13 (1.4%)	12 (3%)	764 (21.7%)	87 (5.5%)	37 (2.1%)	30 (2.7%)	165 (15.2%)	311 (17.1%)
Primary	324 (21.8%)	39 (18.9%)	81 (8.7%)	38 (9.4%)	332 (9.5%)	104 (6.5%)	891 (51.1%)	560 (50.4%)	261 (24.1%)	189 (10.4%)
Secondary	933 (62.7%)	127 (61.7%)	634 (67.8%)	210 (51.7%)	2058 (58.6%)	986 (61.9%)	773 (44.3%)	396 (35.6%)	655 (60.5%)	1179 (64.7%)
> Secondary	118 (7.9%)	30 (14.6%)	207 (22.1%)	146 (36%)	359 (10.2%)	415 (26.1%)	44 (2.5%)	126 (11.3%)	2 (0.2%)	144 (7.9%)

* RESA: Reported ever having sexual activity

	Timor-Leste (2016)		Uganda (2016)		South Africa (2016)		Zambia (2018)		Zimbabwe (2015)	
	RESA*=0 N=1245	RESA*=1 N=467	RESA*=0 N=871	RESA*=1 N=1158	RESA*=0 N=432	RESA*=1 N=1809	RESA*=0 N=1726	RESA*=1 N=3208	RESA*=0 N=1651	RESA*=1 N=1614
**Age Group**										
15-19	771 (61.9%)	95 (20.3%)	730 (83.8%)	506 (43.7%)	345 (79.9%)	359 (19.8%)	1443 (83.6%)	1255 (39.1%)	1320 (80%)	543 (33.6%)
20-24	298 (23.9%)	155 (33.2%)	105 (12.1%)	453 (39.1%)	50 (11.6%)	514 (28.4%)	215 (12.5%)	1204 (37.5%)	248 (15%)	670 (41.5%)
25-29	91 (7.3%)	100 (21.4%)	17 (2%)	142 (12.3%)	18 (4.2%)	344 (19%)	40 (2.3%)	465 (14.5%)	46 (2.8%)	271 (16.8%)
30-34	36 (2.9%)	61 (13.1%)	11 (1.3%)	38 (3.3%)	5 (1.2%)	218 (12.1%)	9 (0.5%)	129 (4%)	22 (1.3%)	83 (5.1%)
35-39	17 (1.4%)	22 (4.7%)	5 (0.6%)	12 (1%)	3 (0.7%)	132 (7.3%)	9 (0.5%)	75 (2.3%)	7 (0.4%)	27 (1.7%)
40-44	15 (1.2%)	9 (1.9%)	1 (0.1%)	2 (0.2%)	2 (0.5%)	102 (5.6%)	5 (0.3%)	34 (1.1%)	5 (0.3%)	13 (0.8%)
45-49	9 (0.7%)	8 (1.7%)	1 (0.1%)	4 (0.3%)	2 (0.5%)	67 (3.7%)	2 (0.1%)	24 (0.7%)	2 (0.1%)	6 (0.4%)
50-54	4 (0.3%)	11 (2.4%)	1 (0.1%)	1 (0.1%)	3 (0.7%)	47 (2.6%)	3 (0.2%)	16 (0.5%)	1 (0.1%)	1 (0.1%)
55-59	4 (0.3%)	6 (1.3%)			4 (0.9%)	26 (1.4%)	0 (0%)	6 (0.2%)		
**Interviewer had survey exp.**	754 (60.6%)	300 (64.2%)	680 (78.1%)	841 (72.6%)	338 (78.2%)	1375 (76%)	1715 (99.4%)	3147 (98.1%)	1140 (69%)	972 (60.2%)
**Rural**	898 (72.1%)	244 (52.2%)	702 (80.6%)	819 (70.7%)	229 (53%)	842 (46.5%)	953 (55.2%)	1874 (58.4%)	1104 (66.9%)	909 (56.3%)
**≥ 10 year age difference**	565 (45.4%)	109 (23.3%)	636 (73%)	515 (44.5%)	289 (66.9%)	475 (26.3%)	1708 (99%)	2981 (92.9%)	1035 (62.7%)	468 (29%)
**Wealth Index**										
1	202 (16.2%)	55 (11.8%)	198 (22.7%)	133 (11.5%)	99 (22.9%)	400 (22.1%)	235 (13.6%)	441 (13.7%)	234 (14.2%)	180 (11.2%)
2	281 (22.6%)	67 (14.3%)	180 (20.7%)	185 (16%)	102 (23.6%)	440 (24.3%)	317 (18.4%)	574 (17.9%)	315 (19.1%)	249 (15.4%)
3	266 (21.4%)	92 (19.7%)	154 (17.7%)	229 (19.8%)	85 (19.7%)	452 (25%)	342 (19.8%)	727 (22.7%)	368 (22.3%)	295 (18.3%)
4	291 (23.4%)	124 (26.6%)	182 (20.9%)	244 (21.1%)	84 (19.4%)	344 (19%)	376 (21.8%)	644 (20.1%)	355 (21.5%)	403 (25%)
5	205 (16.5%)	129 (27.6%)	157 (18%)	367 (31.7%)	62 (14.4%)	173 (9.6%)	456 (26.4%)	822 (25.6%)	379 (23%)	487 (30.2%)
**Respondent education level**										
None	168 (13.5%)	63 (13.5%)	20 (2.3%)	20 (1.7%)	6 (1.4%)	47 (2.6%)	54 (3.1%)	74 (2.3%)	6 (0.4%)	8 (0.5%)
Primary	177 (14.2%)	51 (10.9%)	571 (65.6%)	530 (45.8%)	99 (22.9%)	231 (12.8%)	743 (43%)	1039 (32.4%)	378 (22.9%)	359 (22.2%)
Secondary	851 (68.4%)	261 (55.9%)	250 (28.7%)	442 (38.2%)	322 (74.5%)	1375 (76%)	896 (51.9%)	1831 (57.1%)	1209 (73.2%)	1076 (66.7%)
> Secondary	49 (3.9%)	92 (19.7%)	30 (3.4%)	166 (14.3%)	5 (1.2%)	156 (8.6%)	33 (1.9%)	264 (8.2%)	58 (3.5%)	171 (10.6%)

* RESA: Reported ever having sexual activity

Simple logistic regression models

Table S3. Odds ratios, 95% credible intervals, and posterior probability that the odds are greater than 1 – Women's survey*

	Benin (2017-18)		Burundi (2016-17)		Cameroon (2018)		Ethiopia (2016)	
	Estimate (95% CrI)	PP>1*	Estimate (95% CrI)	PP>1*	Estimate (95% CrI)	PP>1*	Estimate (95% CrI)	PP>1*
Age difference ≥10 years	0.38 (0.33, 0.43)	0	0.32 (0.28, 0.37)	0	0.24 (0.22, 0.27)	0	0.25 (0.16, 0.36)	0
Respondent age	1.47 (1.43, 1.52)	1	1.12 (1.11, 1.13)	1	1.47 (1.43, 1.5)	1	1.19 (1.17, 1.2)	1
Respondent education level	1.21 (1.12, 1.3)	1	0.89 (0.81, 0.98)	0.007	1.68 (1.54, 1.84)	1	1.82 (1.64, 2.02)	1
Wealth Index	1.12 (1.07, 1.17)	1	1.17 (1.11, 1.23)	1	1.22 (1.17, 1.28)	1	1.56 (1.44, 1.7)	1
Interviewer age	0.99 (0.98, 1)	0.128	1 (0.99, 1.02)	0.617	1.01 (1, 1.02)	0.963	1.01 (0.99, 1.04)	0.809
Previous survey experience	1.03 (0.9, 1.18)	0.659	0.79 (0.61, 1.04)	0.047	0.78 (0.7, 0.89)	<0.001	1.62 (1.34, 1.97)	1
Rural residency	0.86 (0.76, 0.98)	0.01	0.49 (0.42, 0.56)	0	0.8 (0.71, 0.89)	<0.001	0.24 (0.2, 0.3)	0
Difference in native lang.	1.35 (1.19, 1.54)	1	0.99 (0.03, 7.93)	0.496	1.21 (1.06, 1.37)	0.997	0.69 (0.58, 0.84)	0
Fieldworker education level	0.83 (0.52, 1.3)	0.206	0.97 (0.79, 1.2)	0.375	0.93 (0.65, 1.32)	0.349	1.32 (1.08, 1.62)	0.997

*Posterior probabilities are the proportion of posterior samples of odds ratios that fall above 1, where either 1 or 0, no samples are below or above 0, respectively

Table S3. Odds ratios, 95% credible intervals, and posterior probability that the odds are greater than 1 – Women's survey (continued)*

	Gambia (2019-20)		Guinea (2018)		Haiti (2017-18)		Liberia (2019-20)	
	Estimate (95% CrI)	PP>1*	Estimate (95% CrI)	PP>1*	Estimate (95% CrI)	PP>1*	Estimate (95% CrI)	PP>1*
Age difference ≥10 years	0.35 (0.28, 0.44)	0	0.31 (0.26, 0.37)	0	0.33 (0.29, 0.37)	0	0.16 (0.12, 0.21)	0
Respondent age	1.26 (1.23, 1.29)	1	1.33 (1.29, 1.37)	1	1.25 (1.23, 1.27)	1	1.86 (1.74, 2.01)	1
Respondent education level	1.09 (0.95, 1.25)	0.878	1.1 (1.02, 1.19)	0.99	1.84 (1.68, 2.02)	1	1.39 (1.22, 1.58)	1
Wealth Index	1.09 (1.01, 1.18)	0.99	1.11 (1.05, 1.18)	>0.999	1.2 (1.16, 1.25)	1	0.92 (0.86, 0.99)	0.014
Interviewer age	1 (0.98, 1.02)	0.509	1.02 (1.01, 1.04)	0.999	0.99 (0.98, 1)	0.01	0.98 (0.96, 0.99)	0.002
Previous survey experience	1.02 (0.81, 1.31)	0.578	1.18 (0.99, 1.41)	0.967	1.22 (1.01, 1.47)	0.981	0.76 (0.59, 0.97)	0.014
Rural residency	0.57 (0.44, 0.73)	0	0.64 (0.54, 0.75)	0	0.62 (0.55, 0.69)	0	0.94 (0.77, 1.14)	0.26
Difference in native lang.	1.33 (1.04, 1.72)	0.989	0 (0, 0)	0	0 (0, 0)	0	0 (0, 0)	0
Fieldworker education level	1.01 (0.92, 1.11)	0.582	1.38 (1.05, 1.84)	0.99	0.93 (0.75, 1.14)	0.23	†	

* Posterior probabilities are the proportion of posterior samples of odds ratios that fall above 1, where either 1 or 0, no samples are below or above 0, respectively
† Model was not possible to build, either because predictor variable did not exist or had no variance.

Table S3. Odds ratios, 95% credible intervals, and posterior probability that the odds are greater than 1 – Women's survey (continued)*

	Mali (2018)		Malawi (2015-16)		Nigeria (2018)		Philippines (2017)	
	Estimate (95% CrI)	PP>1*	Estimate (95% CrI)	PP>1*	Estimate (95% CrI)	PP>1	Estimate (95% CrI)	PP>1*
Age difference ≥10 years	0.51 (0.42, 0.63)	0	0.74 (0.63, 0.86)	<0.001	0.18 (0.16, 0.2)	0	0.3 (0.26, 0.33)	0
Respondent age	1.24 (1.21, 1.28)	1	1.33 (1.3, 1.36)	1	1.35 (1.34, 1.37)	1	1.12 (1.11, 1.13)	1
Respondent education level	1.3 (1.18, 1.44)	1	1.69 (1.54, 1.86)	1	1.85 (1.74, 1.96)	1	1.64 (1.48, 1.82)	1
Wealth Index	1.3 (1.21, 1.39)	1	1.11 (1.07, 1.16)	1	1.18 (1.14, 1.22)	1	1.15 (1.1, 1.2)	1
Interviewer age	1.03 (1.01, 1.04)	0.999	1.01 (1, 1.02)	0.97	1.01 (1, 1.02)	>0.999	0.99 (0.98, 0.99)	<0.001
Previous survey experience	1.77 (1.34, 2.36)	>0.999	1.14 (1.02, 1.28)	0.986	0.99 (0.89, 1.11)	0.45	0.83 (0.69, 0.99)	0.021
Rural residency	0.7 (0.57, 0.85)	<0.001	0.7 (0.62, 0.8)	0	0.99 (0.92, 1.07)	0.421	0.61 (0.54, 0.69)	0
Difference in native lang.	†		0.96 (0.84, 1.11)	0.305	0.82 (0.75, 0.89)	0	0.78 (0.65, 0.94)	0.004
Fieldworker education level	0.95 (0.77, 1.18)	0.332	0.86 (0.76, 0.97)	0.008	†		0.43 (0.28, 0.64)	0

* Posterior probabilities are the proportion of posterior samples of odds ratios that fall above 1, where either 1 or 0, no samples are below or above 0, respectively
† Model was not possible to build, either because predictor variable did not exist or had no variance.

Table S3. Odds ratios, 95% credible intervals, and posterior probability that the odds are greater than 1 – Women's survey (continued)*

	Rwanda (2019-20)		Sierra Leone (2019)		Timor-Leste (2016)		Uganda (2016)	
	Estimate (95% CrI)	PP>1*	Estimate (95% CrI)	PP>1*	Estimate (95% CrI)	PP>1*	Estimate (95% CrI)	PP>1
Age difference ≥10 years	0.33 (0.29, 0.37)	0	0.12 (0.1, 0.14)	0	0.19 (0.11, 0.31)	0	0.32 (0.28, 0.36)	0
Respondent age	1.2 (1.18, 1.21)	1	1.79 (1.72, 1.86)	1	1.12 (1.1, 1.14)	1	1.33 (1.3, 1.36)	1
Respondent education level	0.67 (0.61, 0.73)	0	1.18 (1.1, 1.27)	>0.999	0.74 (0.62, 0.9)	0.002	1.92 (1.76, 2.1)	1
Wealth Index	0.98 (0.94, 1.02)	0.135	0.99 (0.95, 1.04)	0.375	0.91 (0.82, 1.02)	0.057	1.23 (1.18, 1.29)	1
Interviewer age	1 (0.99, 1.01)	0.682	0.99 (0.98, 1.01)	0.206	1.01 (0.98, 1.05)	0.791	0.99 (0.98, 1)	0.003
Previous survey experience	0.97 (0.87, 1.08)	0.268	0.83 (0.71, 0.96)	0.007	0.67 (0.48, 0.92)	0.006	0.82 (0.72, 0.93)	0.001
Rural residency	0.68 (0.6, 0.76)	0	0.93 (0.82, 1.05)	0.117	1.19 (0.86, 1.63)	0.851	0.63 (0.55, 0.72)	0
Difference in native lang.	†		†		1.75 (1.28, 2.39)	>0.999	1.19 (1.05, 1.34)	0.997
Fieldworker education level	1.06 (0.85, 1.31)	0.684	0.97 (0.76, 1.23)	0.393	1.26 (0.85, 1.93)	0.871	1.01 (0.86, 1.19)	0.543

* Posterior probabilities are the proportion of posterior samples of odds ratios that fall above 1, where either 1 or 0, no samples are below or above 0, respectively
† Model was not possible to build, either because predictor variable did not exist or had no variance.

Table S3. Odds ratios, 95% credible intervals, and posterior probability that the odds are greater than 1 – Women's survey (continued)*

	**South Africa (2016)**		**Zambia (2018)**		**Zimbabwe (2015)**	
	Estimate (95% CrI)	PP>1*	Estimate (95% CrI)	PP>1*	Estimate (95% CrI)	PP>1*
Age difference ≥10 years	0.11 (0.09, 0.13)	0	0.31 (0.26, 0.37)	0	0.25 (0.2, 0.3)	0
Respondent age	1.44 (1.4, 1.48)	1	1.39 (1.35, 1.42)	1	1.24 (1.21, 1.27)	1
Respondent education level	1.16 (1, 1.34)	0.978	1.43 (1.3, 1.58)	1	1.03 (0.86, 1.24)	0.636
Wealth Index	0.85 (0.81, 0.9)	0	0.92 (0.88, 0.96)	<0.001	1.03 (0.97, 1.1)	0.82
Interviewer age	0.98 (0.98, 0.99)	0.001	1 (0.99, 1)	0.182	0.98 (0.95, 1)	0.029
Previous survey experience	1.16 (0.95, 1.41)	0.933	1.04 (0.92, 1.18)	0.723	0.49 (0.41, 0.58)	0
Rural residency	1.14 (1, 1.31)	0.972	1.06 (0.93, 1.2)	0.812	0.79 (0.66, 0.94)	0.005
Difference in native lang.	0 (0, 0)	0	1.08 (0.95, 1.23)	0.891	0 (0, 0)	0
Fieldworker education level	1.11 (0.96, 1.28)	0.911	0.49 (0.33, 0.72)	<0.001	1.31 (0.36, 6.39)	0.654

* Posterior probabilities are the proportion of posterior samples of odds ratios that fall above 1, where either 1 or 0, no samples are below or above 0, respectively
† Model was not possible to build, either because predictor variable did not exist or had no variance.

Table S4. Odds ratios, 95% credible intervals, and posterior probability that the odds are greater than 1 – Men's survey*

	**Benin (2017-18)**		**Burundi (2016-17)**		**Cameroon (2018)**		**Ethiopia (2016)**	
	Estimate (95% CrI)	PP>1	Estimate (95% CrI)	PP>1*	Estimate (95% CrI)	PP>1*	Estimate (95% CrI)	PP>1*
Age difference ≥10 years	0.16 (0.14, 0.19)	0	0.33 (0.27, 0.4)	0	0.22 (0.19, 0.25)	0	0.23 (0.19, 0.27)	0
Respondent age	1.5 (1.45, 1.55)	1	1.14 (1.12, 1.16)	1	1.33 (1.31, 1.37)	1	1.22 (1.21, 1.24)	1
Respondent education level	1.24 (1.14, 1.34)	1	1 (0.88, 1.13)	0.479	1.9 (1.71, 2.12)	1	2.11 (1.95, 2.28)	1
Wealth Index	1.15 (1.1, 1.22)	1	1.17 (1.1, 1.25)	1	1.52 (1.43, 1.6)	1	1.42 (1.35, 1.49)	1
Interviewer age	0.96 (0.95, 0.98)	<0.001	0.98 (0.96, 0.99)	<0.001	1 (0.99, 1.02)	0.623	0.99 (0.98, 1)	0.123
Previous survey experience	0.7 (0.6, 0.82)	0	0.84 (0.63, 1.14)	0.124	0.77 (0.66, 0.9)	<0.001	0.68 (0.55, 0.85)	<0.001
Rural residency	0.77 (0.67, 0.89)	<0.001	0.54 (0.45, 0.64)	0	0.61 (0.53, 0.7)	0	0.31 (0.27, 0.35)	0
Difference in native lang.	1.22 (1.05, 1.42)	0.995	7.55 (1.52, 59.02)	0.993	1.65 (1.4, 1.94)	1	1.13 (0.88, 1.47)	0.827
Fieldworker education level	†		†		1.76 (1.35, 2.29)	1	0.94 (0.82, 1.09)	0.209

* Posterior probabilities are the proportion of posterior samples of odds ratios that fall above 1, where either 1 or 0, no samples are below or above 0, respectively
† Model was not possible to build, either because predictor variable did not exist or had no variance.

Table S4. Odds ratios, 95% credible intervals, and posterior probability that the odds are greater than 1 – Men's survey (continued)*

	**Gambia (2019-20)**		**Guinea (2018)**		**Haiti (2017-18)**		**Liberia (2019-20)**	
	Estimate (95% CrI)	PP>1*	Estimate (95% CrI)	PP>1	Estimate (95% CrI)	PP>1*	Estimate (95% CrI)	PP>1*
Age difference ≥10 years	0.35 (0.3, 0.42)	0	0.44 (0.35, 0.54)	0	0.29 (0.25, 0.34)	0	0.13 (0.08, 0.18)	0
Respondent age	1.22 (1.19, 1.25)	1	1.25 (1.22, 1.29)	1	1.33 (1.3, 1.37)	1	1.55 (1.47, 1.63)	1
Respondent education level	1.3 (1.18, 1.43)	1	1.34 (1.22, 1.48)	1	2.47 (2.22, 2.75)	1	2.72 (2.32, 3.21)	1
Wealth Index	1.17 (1.1, 1.23)	1	1.17 (1.09, 1.26)	1	1.41 (1.34, 1.48)	1	1.17 (1.08, 1.27)	>0.999
Interviewer age	1.02 (1, 1.03)	0.971	1.02 (1, 1.03)	0.987	0.99 (0.99, 1)	0.034	1.01 (0.99, 1.03)	0.838
Previous survey experience	1.08 (0.89, 1.31)	0.778	0.87 (0.66, 1.14)	0.151	1.02 (0.87, 1.18)	0.577	0.78 (0.6, 1)	0.027
Rural residency	0.58 (0.49, 0.69)	0	0.65 (0.54, 0.78)	0	0.46 (0.4, 0.54)	0	0.6 (0.49, 0.74)	0
Difference in native lang.	0.96 (0.81, 1.13)	0.304	†		†		†	
Fieldworker education level	0.87 (0.75, 1.02)	0.041	†		0.8 (0.53, 1.17)	0.127	0.5 (0.28, 0.87)	0.006

* Posterior probabilities are the proportion of posterior samples of odds ratios that fall above 1, where either 1 or 0, no samples are below or above 0, respectively
† Model was not possible to build, either because predictor variable did not exist or had no variance.

Table S4. Odds ratios, 95% credible intervals, and posterior probability that the odds are greater than 1 – Men's survey (continued)*

	Mali (2018)		Malawi (2015-16)		Myanmar (2015-16)		Nepal (2016)	
	Estimate (95% CrI)	PP>1*	Estimate (95% CrI)	PP>1*	Estimate (95% CrI)	PP>1	Estimate (95% CrI)	PP>1*
Age difference ≥10 years	0.31 (0.25, 0.38)	0	0.5 (0.42, 0.6)	0	0.52 (0.31, 0.82)	0.002	0.4 (0.31, 0.52)	0
Respondent age	1.27 (1.23, 1.31)	1	1.28 (1.24, 1.32)	1	1.07 (1.05, 1.09)	1	1.25 (1.2, 1.29)	1
Respondent education level	1.59 (1.43, 1.77)	1	1.57 (1.37, 1.81)	1	1.36 (1.1, 1.7)	0.998	1.29 (1.07, 1.55)	0.996
Wealth Index	1.25 (1.17, 1.35)	1	1 (0.94, 1.06)	0.47	1.28 (1.14, 1.43)	1	1.03 (0.94, 1.12)	0.724
Interviewer age	1 (0.98, 1.03)	0.605	0.99 (0.98, 0.99)	<0.001	1.01 (0.99, 1.02)	0.803	1 (0.97, 1.02)	0.46
Previous survey experience	1.17 (0.93, 1.48)	0.905	0.84 (0.72, 0.99)	0.017	0.8 (0.6, 1.07)	0.068	1.28 (0.73, 2.32)	0.804
Rural residency	0.75 (0.61, 0.92)	0.003	0.87 (0.72, 1.04)	0.068	0.57 (0.43, 0.77)	<0.001	1.29 (1, 1.65)	0.977
Difference in native lang.	†		0.91 (0.76, 1.08)	0.133	†		0.9 (0.71, 1.14)	0.196
Fieldworker education level	0.7 (0.5, 0.99)	0.021	0.97 (0.84, 1.12)	0.333	†		†	

* Posterior probabilities are the proportion of posterior samples of odds ratios that fall above 1, where either 1 or 0, no samples are below or above 0, respectively
† Model was not possible to build, either because predictor variable did not exist or had no variance.

Table S4. Odds ratios, 95% credible intervals, and posterior probability that the odds are greater than 1 – Men's survey (continued)*

	Nigeria (2018)		Rwanda (2019-20)		Sierra Leone (2019)		Timor-Leste (2016)	
	Estimate (95% CrI)	PP>1*	Estimate (95% CrI)	PP>1*	Estimate (95% CrI)	PP>1*	Estimate (95% CrI)	PP>1
Age difference ≥10 years	0.25 (0.22, 0.29)	0	0.32 (0.27, 0.38)	0	0.19 (0.16, 0.23)	0	0.37 (0.29, 0.46)	0
Respondent age	1.22 (1.2, 1.23)	1	1.25 (1.22, 1.28)	1	1.7 (1.63, 1.77)	1	1.12 (1.1, 1.14)	1
Respondent education level	2.09 (1.93, 2.28)	1	1.22 (1.09, 1.38)	>0.999	1.3 (1.19, 1.42)	1	1.37 (1.19, 1.58)	1
Wealth Index	1.55 (1.48, 1.62)	1	1.2 (1.13, 1.27)	1	1.2 (1.13, 1.26)	1	1.27 (1.17, 1.38)	1
Interviewer age	1 (0.99, 1)	0.157	0.99 (0.98, 1)	0.032	1 (0.98, 1.02)	0.515	1.03 (1.01, 1.06)	0.998
Previous survey experience	0.98 (0.83, 1.15)	0.403	0.6 (0.51, 0.71)	0	0.89 (0.72, 1.1)	0.135	1.17 (0.94, 1.46)	0.917
Rural residency	0.68 (0.6, 0.77)	0	0.5 (0.43, 0.6)	0	0.64 (0.55, 0.75)	0	0.42 (0.34, 0.53)	0
Difference in native lang.	1.09 (0.96, 1.23)	0.914	†		†		0.43 (0.26, 0.68)	<0.001
Fieldworker education level	47.64 (8.82, 1376.81)	1	†		0.95 (0.76, 1.16)	0.302	0.65 (0.5, 0.86)	0.001

* Posterior probabilities are the proportion of posterior samples of odds ratios that fall above 1, where either 1 or 0, no samples are below or above 0, respectively
† Model was not possible to build, either because predictor variable did not exist or had no variance.

Table S4. Odds ratios, 95% credible intervals, and posterior probability that the odds are greater than 1 – Men's survey (continued)*

	Uganda (2016)		South Africa (2016)		Zambia (2018)		Zimbabwe (2015)	
	Estimate (95% CrI)	PP>1*	Estimate (95% CrI)	PP>1*	Estimate (95% CrI)	PP>1*	Estimate (95% CrI)	PP>1*
Age difference ≥10 years	0.3 (0.24, 0.36)	0	0.18 (0.14, 0.22)	0	0.14 (0.08, 0.21)	0	0.24 (0.21, 0.28)	0
Respondent age	1.31 (1.27, 1.35)	1	1.28 (1.25, 1.33)	1	1.34 (1.31, 1.38)	1	1.31 (1.28, 1.35)	1
Respondent education level	2.03 (1.77, 2.34)	1	1.61 (1.34, 1.94)	1	1.6 (1.46, 1.76)	1	1.3 (1.14, 1.48)	>0.999
Wealth Index	1.32 (1.23, 1.4)	1	0.95 (0.87, 1.03)	0.093	0.98 (0.94, 1.03)	0.225	1.16 (1.1, 1.22)	1
Interviewer age	0.97 (0.96, 0.98)	<0.001	1 (0.99, 1.02)	0.719	0.98 (0.98, 0.99)	0	0.98 (0.96, 0.99)	<0.001
Previous survey experience	0.75 (0.61, 0.92)	0.003	0.88 (0.68, 1.13)	0.155	0.32 (0.16, 0.59)	<0.001	0.68 (0.59, 0.78)	0
Rural residency	0.58 (0.47, 0.72)	0	0.77 (0.63, 0.96)	0.008	1.14 (1.01, 1.28)	0.985	0.64 (0.55, 0.74)	0
Difference in native lang.	1.01 (0.84, 1.23)	0.559	†		1.07 (0.95, 1.2)	0.851	†	
Fieldworker education level	0.78 (0.63, 0.96)	0.01	0.85 (0.68, 1.05)	0.062	0.92 (0.8, 1.06)	0.121	0.63 (0.47, 0.85)	0.001

* Posterior probabilities are the proportion of posterior samples of odds ratios that fall above 1, where either 1 or 0, no samples are below or above 0, respectively
† Model was not possible to build, either because predictor variable did not exist or had no variance.

Full models

Table S5. Log odds, 95% credible intervals, and posterior probability that log odds are greater than 0 from full model of ever reporting sexual intercourse among never-unioned women

	Benin (2017-18)		Burundi (2016-17)		Cameroon (2018)		Ethiopia (2016)		Gambia (2019-20)	
	Estimate (95% CrI)	Posterior Prob. > 0	Estimate (95% CrI)	Posterior Prob. > 0	Estimate (95% CrI)	Posterior Prob. > 0	Estimate (95% CrI)	Posterior Prob. > 0	Estimate (95% CrI)	Posterior Prob. > 0
Intercept	-1.84 (-2.43,-1.25)		-1.9 (-2.67,-1.16)		-2.31 (-3.22,-1.41)		-3.8 (-4.81,-2.81)		-2.8 (-3.6,-2.04)	
**Level 1 (Respondent)**										
Age difference ≥10 years	-0.42 (-0.73, -0.12)	0.003	-0.26 (-0.52, 0)	0.027	-0.44 (-0.75, -0.14)	0.002	-0.5 (-1.05, 0.03)	0.032	-0.27 (-0.77, -0.21)	0.138
Respondent age*	0.42 (0.38,0.46)	1	0.11 (0.09,0.13)	1	0.43 (0.39,0.46)	1	0.17 (0.15,0.19)	1	0.25 (0.22,0.29)	1
Respondent edu. Level	0.04 (-0.06,0.14)	0.778	-0.07 (-0.18,0.04)	0.106	0.27 (0.11,0.44)	> 0.999	0.2 (0.07,0.34)	0.999	-0.11 (-0.28,0.06)	0.101
Wealth Index†	0.02 (-0.05,0.1)	0.709	0.05 (-0.02,0.13)	0.91	-0.17 (-0.27,-0.06)	< 0.001	-0.04 (-0.19,0.12)	0.321	-0.32 (-0.46,-0.19)	0
**Level 2a (Interviewer)**										
Interviewer age‡	0 (-0.04,0.04)	0.472	0.01 (-0.02,0.03)	0.681	0.04 (-0.02,0.1)	0.93	0.01 (-0.06,0.08)	0.596	0 (-0.04,0.04)	0.504
Interviewer survey experience	0.12 (-0.29,0.52)	0.715	-0.26 (-0.8,0.3)	0.176	-0.49 (-1.12,0.15)	0.066	0.05 (-0.38,0.47)	0.588	0.03 (-0.38,0.44)	0.55
**Level 2b (Survey cluster)**										
Rural	0.05 (-0.16,0.26)	0.672	-0.69 (-0.93,-0.45)	0	0.21 (-0.08,0.5)	0.921	-0.89 (-1.37,-0.43)	< 0.001	-0.72 (-1.19,-0.28)	< 0.001
**Variance Components**										
Cluster-specific variance	0.263 (0.128 , 0.441)		0.253 (0.132 , 0.41)		0.481 (0.305 , 0.705)		0.704 (0.388 , 1.131)		0.475 (0.204 , 0.856)	
Interviewer-specific variance	0.513 (0.317,0.807)		0.224 (0.12 , 0.385)		1.288 (0.825 , 1.994)		0.555 (0.268 , 0.982)		0.079 (0.001 , 0.276)	
# of clusters	545		554		428		619		280	
# of fieldworkers	73		75		68		131		51	
# of observations	3897		6091		4915		4250		3226	

* Respondent age in years centered at 15
† Wealth index centered at 2 of 5, with 5 being highest relative wealth
‡ Interviewer age in years centered at 18

Table S5. Log odds, 95% credible intervals, and posterior probability that log odds are greater than 0 from full model of ever reporting sexual intercourse among never-unioned women (Continued)

	Guinea (2018)		Haiti (2017-18)		Liberia (2019-20)		Mali (2018)		Malawi (2015-16)	
	Estimate (95% CrI)	Posterior Prob. > 0	Estimate (95% CrI)	Posterior Prob. > 0	Estimate (95% CrI)	Posterior Prob. > 0	Estimate (95% CrI)	Posterior Prob. > 0	Estimate (95% CrI)	Posterior Prob. > 0
Intercept	-2.29 (-3.45,-1.16)		-0.85 (-1.29,-0.43)		0.4 (-0.42,1.24)		-2.75 (-3.81,-1.72)		-1.41 (-1.95,-0.87)	
**Level 1 (Respondent)**										
Age difference ≥10 years	-0.28 (-0.65, 0.1)	0.071	-0.07 (-0.28, 0.14)	0.249	-0.6 (-1.15,-0.04)	0.018	-0.07 (-0.53, 0.39)	0.379	-0.28 (-0.65, 0.07)	0.059
Respondent age*	0.35 (0.3,0.39)	1	0.21 (0.19,0.23)	1	0.66 (0.58,0.75)	1	0.27 (0.23,0.32)	1	0.31 (0.29,0.35)	1
Respondent edu. Level	-0.08 (-0.2,0.05)	0.112	0.32 (0.2,0.44)	1	0.33 (0.1,0.57)	0.997	0 (-0.16,0.15)	0.485	-0.09 (-0.23,0.06)	0.115
Wealth Index†	-0.19 (-0.32,-0.05)	0.003	-0.12 (-0.19,-0.05)	< 0.001	-0.38 (-0.52,-0.25)	0	0.12 (-0.03,0.27)	0.943	-0.03 (-0.09,0.03)	0.153
**Level 2a (Interviewer)**										
Interviewer age‡	0.03 (-0.04,0.1)	0.817	-0.01 (-0.03,0)	0.026	-0.01 (-0.05,0.03)	0.278	0.02 (-0.04,0.08)	0.778	0.03 (-0.01,0.06)	0.939
Interviewer survey experience	0.11 (-0.7,0.92)	0.609	0.18 (-0.14,0.5)	0.869	-0.44 (-0.91,0.02)	0.029	0.3 (-0.5,1.11)	0.769	0.09 (-0.19,0.37)	0.734
**Level 2b (Survey cluster)**										
Rural	-0.15 (-0.52,0.22)	0.214	-0.43 (-0.63,-0.23)	0	0.05 (-0.33,0.44)	0.611	-0.05 (-0.54,0.45)	0.43	-0.12 (-0.32,0.07)	0.115
**Variance Components**										
Cluster-specific variance	0.219 (0.046 , 0.47)		0.242 (0.154 , 0.35)		0.382 (0.13 , 0.74)		0.558 (0.26 , 0.984)		0.173 (0.065 , 0.311)	
Interviewer-specific variance	1.651 (1.009 , 2.67)		0.024 (0.001 , 0.073)		0.204 (0.033 , 0.505)		0.978 (0.525 , 1.696)		0.36 (0.238 , 0.526)	
# of clusters	376		450		319		307		810	
# of fieldworkers	61		55		54		71		140	
# of observations	2645		5760		2621		1816		4993	

* Respondent age in years centered at 15
† Wealth index centered at 2 of 5, with 5 being highest relative wealth
‡ Interviewer age in years centered at 18

Table S5. Log odds, 95% credible intervals, and posterior probability that log odds are greater than 0 from full model of ever reporting sexual intercourse among never-unioned women (Continued)

	Nigeria (2018)		Philippines (2017)		Rwanda (2019-20)		Sierra Leone (2019)		Timor-Leste (2016)	
	Estimate (95% CrI)	Posterior Prob. > 0	Estimate (95% CrI)	Posterior Prob. > 0	Estimate (95% CrI)	Posterior Prob. > 0	Estimate (95% CrI)	Posterior Prob. > 0	Estimate (95% CrI)	Posterior Prob. > 0
Intercept	-2.64 (-3.97,-1.34)		-2.84 (-3.37,-2.3)		-1.11 (-1.47,-0.76)		-0.56 (-1.36,0.23)		-6.51 (-7.94,-5.17)	
**Level 1 (Respondent)**										
Age difference ≥10 years	0.36 (0.1, 0.61)	0.997	-0.09 (-0.34, 0.15)	0.227	-0.25 (-0.46,-0.04)	0.010	-0.33 (-0.67,0.02)	0.031	-1.41 (-2.11,0.75)	0
Respondent age*	0.34 (0.32,0.36)	1	0.12 (0.11,0.13)	1	0.17 (0.15,0.18)	1	0.68 (0.63,0.73)	1	0.13 (0.1,0.16)	1
Respondent edu. Level	0.07 (-0.03,0.17)	0.928	0.34 (0.22,0.47)	1	-0.36 (-0.47,-0.25)	0	0.22 (0.08,0.35)	> 0.999	0.03 (-0.22,0.27)	0.58
Wealth Index†	-0.2 (-0.27,-0.14)	0	-0.15 (-0.21,-0.08)	0	-0.03 (-0.08,0.03)	0.145	-0.24 (-0.34,-0.14)	0	-0.15 (-0.33,0.03)	0.052
**Level 2a (Interviewer)**										
Interviewer age‡	0.01 (-0.05,0.06)	0.574	-0.02 (-0.04,0)	0.007	0.01 (-0.01,0.02)	0.888	-0.02 (-0.07,0.02)	0.149	0.08 (0,0.16)	0.978
Interviewer survey experience	0 (-1.05,1.03)	0.497	-0.08 (-0.46,0.31)	0.352	-0.03 (-0.2,0.13)	0.337	-0.22 (-0.72,0.28)	0.197	-0.42 (-1.12,0.28)	0.118
**Level 2b (Survey cluster)**										
Rural	0.1 (-0.06,0.26)	0.894	-0.52 (-0.71,-0.33)	0	-0.4 (-0.57,-0.22)	0	0.08 (-0.21,0.37)	0.7	-0.19 (-0.79,0.39)	0.264
**Variance Components**										
Cluster-specific variance	0.319 (0.22 , 0.435)		0.309 (0.163 , 0.49)		0.105 (0.031 , 0.202)		0.343 (0.188 , 0.541)		1.065 (0.353 , 2.19)	
Interviewer-specific variance	3.441 (2.422 , 4.906)		0.458 (0.314 , 0.642)		0.012 (0 , 0.054)		0.466 (0.27 , 0.775)		1.252 (0.635 , 2.312)	
# of clusters	1328		1230		500		557		452	
# of fieldworkers	116		262		56		66		75	
# of observations	10669		8652		5961		4966		4412	

* Respondent age in years centered at 15
† Wealth index centered at 2 of 5, with 5 being highest relative wealth
‡ Interviewer age in years centered at 18

Table S5. Log odds, 95% credible intervals, and posterior probability that log odds are greater than 0 from full model of ever reporting sexual intercourse among never-unioned women (Continued)

	**Uganda (2016)**		**South Africa (2016)**		**Zambia (2018)**		**Zimbabwe (2015)**	
	Estimate (95% CrI)	Posterior Prob. > 0	Estimate (95% CrI)	Posterior Prob. > 0	Estimate (95% CrI)	Posterior Prob. > 0	Estimate (95% CrI)	Posterior Prob. > 0
Intercept	-1.63 (-2.08,-1.18)		-0.66 (-1.43,0.1)		-0.47 (-1,0.06)		-2.36 (-3.43,-1.33)	
**Level 1 (Respondent)**								
Age difference ≥10 years	-0.37 (-0.6, 0.14)	< 0.001	-0.32 (-0.64,0)	0.025	0.14 (-0.18, 0.47)	0.07	-0.92 (-1.32, 0.53)	0
Respondent age*	0.26 (0.23,0.29)	1	0.36 (0.33,0.4)	1	0.4 (0.36,0.43)	1	0.21 (0.18,0.24)	1
Respondent edu. Level	0.22 (0.1,0.34)	> 0.999	0.26 (-0.01,0.53)	0.972	0.07 (-0.09,0.23)	0.793	-0.27 (-0.53,-0.01)	0.02
Wealth Index†	0.01 (-0.06,0.08)	0.627	-0.35 (-0.44,-0.25)	0	-0.26 (-0.35,-0.17)	0	-0.14 (-0.29,0.01)	0.035
**Level 2a (Interviewer)**								
Interviewer age‡	0 (-0.03,0.03)	0.5	-0.01 (-0.04,0.01)	0.152	-0.01 (-0.03,0.01)	0.21	0.08 (0.02,0.14)	0.992
Interviewer survey experience	-0.23 (-0.52,0.06)	0.06	0.14 (-0.26,0.55)	0.754	-0.04 (-0.41,0.34)	0.424	-0.42 (-0.9,0.08)	0.05
**Level 2b (Survey cluster)**								
Rural	0.08 (-0.12,0.27)	0.778	-0.16 (-0.42,0.09)	0.103	0.1 (-0.14,0.34)	0.79	-0.05 (-0.5,0.39)	0.409
**Variance Components**								
Cluster-specific variance	0.155 (0.056 , 0.283)		0.079 (0.001 , 0.265)		0.26 (0.13 , 0.428)		0.584 (0.305 , 0.967)	
Interviewer-specific variance	0.229 (0.132 , 0.374)		0.261 (0.142 , 0.438)		0.349 (0.206 , 0.57)		0.441 (0.215 , 0.819)	
# of clusters	685		687		537		391	
# of fieldworkers	86		91		66		58	
# of observations	4567		5134		4105		2386	

* Respondent age in years centered at 15
† Wealth index centered at 2 of 5, with 5 being highest relative wealth
‡ Interviewer age in years centered at 18

Table S6. Log odds, 95% credible intervals, and posterior probability that log odds are greater than 0 from full model of ever reporting sexual intercourse among never-unioned men

	**Benin (2017-18)**		**Burundi (2016-17)**		**Cameroon (2018)**		**Ethiopia (2016)**		**Gambia (2019-20)**	
	Estimate (95% CrI)	Posterior Prob. > 0	Estimate (95% CrI)	Posterior Prob. > 0	Estimate (95% CrI)	Posterior Prob. > 0	Estimate (95% CrI)	Posterior Prob. > 0	Estimate (95% CrI)	Posterior Prob. > 0
Intercept	-2.23 (-3.2,-1.26)		-1.88 (-4.16,0.33)		-2.87 (-4.49,-1.26)		-3.29 (-4.46,-2.13)		-2.05 (-2.72,-1.37)	
**Level 1 (Respondent)**										
Age difference ≥10 years	-0.65 (-1,-0.31)	< 0.001	0.04 (-0.31, 0.39)	0.406	-0.44 (-0.78, 0.1)	0.006	-0.84 (-1.19, -0.5)	0	-0.38 (-0.71,-0.07)	0.009
Respondent age*	0.44 (0.39,0.49)	1	0.16 (0.13,0.19)	1	0.32 (0.28,0.36)	1	0.19 (0.17,0.21)	1	0.18 (0.16,0.21)	1
Respondent edu. Level	0.25 (0.12,0.39)	> 0.999	-0.27 (-0.42,-0.13)	< 0.001	0.38 (0.22,0.55)	1	0.3 (0.19,0.42)	1	0.15 (0.03,0.26)	0.994
Wealth Index†	0 (-0.1,0.1)	0.48	0.05 (-0.04,0.14)	0.849	0.15 (0.04,0.27)	0.995	0.09 (0,0.19)	0.97	-0.02 (-0.12,0.08)	0.349
**Level 2a (Interviewer)**										
Interviewer age‡	-0.01 (-0.08,0.06)	0.35	0.01 (-0.05,0.08)	0.668	0.02 (-0.08,0.12)	0.642	0.02 (-0.03,0.07)	0.811	0.03 (-0.01,0.07)	0.921
Interviewer survey experience	-0.28 (-1.06,0.5)	0.235	0.13 (-1.91,2.16)	0.552	-0.25 (-1.26,0.77)	0.305	-0.59 (-1.62,0.43)	0.123	0.11 (-0.42,0.67)	0.667
**Level 2b (Survey cluster)**										
Rural	0.03 (-0.23,0.3)	0.595	-0.46 (-0.77,-0.17)	< 0.001	0.26 (-0.02,0.54)	0.965	-0.39 (-0.73,-0.06)	0.011	-0.32 (-0.65,0.01)	0.027
**Variance Components**										
Cluster-specific variance	0.394 (0.17 , 0.702)		0.274 (0.109 , 0.499)		0.19 (0.035 , 0.406)		0.58 (0.367 , 0.851)		0.22 (0.081 , 0.417)	
Interviewer-specific variance	0.788 (0.41 , 1.469)		0.917 (0.441 , 1.859)		1.34 (0.759 , 2.386)		1.193 (0.732 , 1.915)		0.137 (0.038 , 0.374)	
# of clusters	545		499		427		619		279	
# of fieldworkers	37		31		34		58		22	
# of observations	2906		2825		3380		4600		2384	

* Respondent age in years centered at 15
† Wealth index centered at 2 of 5, with 5 being highest relative wealth
‡ Interviewer age in years centered at 18

Table S6. Log odds, 95% credible intervals, and posterior probability that log odds are greater than 0 from full model of ever reporting sexual intercourse among never-unioned men (Continued)

	**Guinea (2018)**		**Haiti (2017-18)**		**Liberia (2019-20)**		**Mali (2018)**		**Malawi (2015-16)**	
	Estimate (95% CrI)	Posterior Prob. > 0	Estimate (95% CrI)	Posterior Prob. > 0	Estimate (95% CrI)	Posterior Prob. > 0	Estimate (95% CrI)	Posterior Prob. > 0	Estimate (95% CrI)	Posterior Prob. > 0
Intercept	-2.04 (-3.75,-0.37)		-1.61 (-2.1,-1.13)		-1.86 (-3.21,-0.53)		-3.44 (-5.4,-1.55)		-0.75 (-1.36,-0.14)	
**Level 1 (Respondent)**										
Age difference ≥10 years	0.39 (-0.1, 0.9)	0.938	-0.43 (-0.72, -0.15)	0.001	0.22 (-0.85, 0.4)	0.246	-0.5 (-0.99, -0.02)	0.020	-0.65 (-1.04, -0.28)	< 0.001
Respondent age*	0.32 (0.28,0.37)	1	0.25 (0.22,0.28)	1	0.42 (0.35,0.49)	1	0.34 (0.28,0.4)	1	0.24 (0.2,0.27)	1
Respondent edu. Level	0.21 (0.05,0.36)	0.996	0.89 (0.74,1.05)	1	0.93 (0.68,1.2)	1	0.29 (0.12,0.47)	> 0.999	0.13 (-0.06,0.32)	0.913
Wealth Index†	0.02 (-0.13,0.16)	0.589	0.09 (0,0.17)	0.978	-0.05 (-0.19,0.09)	0.24	0.07 (-0.09,0.24)	0.802	-0.08 (-0.16,0)	0.019
**Level 2a (Interviewer)**										
Interviewer age‡	0.02 (-0.05,0.09)	0.722	0 (-0.02,0.02)	0.473	0 (-0.06,0.06)	0.509	0.03 (-0.1,0.16)	0.662	0.01 (-0.01,0.04)	0.797
Interviewer survey experience	-0.41 (-1.93,1.08)	0.295	0.09 (-0.27,0.45)	0.693	-0.05 (-0.77,0.66)	0.444	0.49 (-0.88,1.9)	0.765	-0.08 (-0.48,0.31)	0.347
**Level 2b (Survey cluster)**										
Rural	0.02 (-0.38,0.43)	0.541	-0.28 (-0.52,-0.04)	0.011	-0.32 (-0.71,0.06)	0.051	-0.41 (-0.93,0.12)	0.066	-0.04 (-0.29,0.22)	0.387
**Variance Components**										
Cluster-specific variance	0.115 (0.001 , 0.411)		0.135 (0.028 , 0.28)		0.321 (0.033 , 0.749)		0.264 (0.008 , 0.73)		0.055 (0 , 0.24)	
Interviewer-specific variance	2.416 (1.373 , 4.237)		0.078 (0.019 , 0.195)		0.324 (0.106 , 0.773)		2.676 (1.469 , 4.742)		0.319 (0.165 , 0.565)	
# of clusters										
# of fieldworkers	363		450		311		322		757	
# of observations	39		30		29		45		67	
Intercept	1761		4608		1543		1580		2666	

* Respondent age in years centered at 15
† Wealth index centered at 2 of 5, with 5 being highest relative wealth
‡ Interviewer age in years centered at 18

Table S6. Log odds, 95% credible intervals, and posterior probability that log odds are greater than 0 from full model of ever reporting sexual intercourse among never-unioned men (Continued)

	Myanmar (2015-16)		Nepal (2016)		Nigeria (2018)		Rwanda (2019-20)		Sierra Leone (2019)	
	Estimate (95% CrI)	Posterior Prob. > 0	Estimate (95% CrI)	Posterior Prob. > 0	Estimate (95% CrI)	Posterior Prob. > 0	Estimate (95% CrI)	Posterior Prob. > 0	Estimate (95% CrI)	Posterior Prob. > 0
Intercept	-4.45 (-5.84,-3.2)		-3.4 (-5.27,-1.63)		-3.6 (-5.42,-1.78)		-0.95 (-1.67,-0.23)		-2.67 (-3.85,-1.54)	
**Level 1 (Respondent)**										
Age difference ≥10 years	-1.23 (-2.26, -0.33)	0.003	-0.59 (-1.08, -0.11)	0.007	0.09 (-0.24, 0.42)	0.699	-0.23 (-0.57, 0.11)	0.091	-0.07 (-0.49, 0.34)	0.361
Respondent age*	0.07 (0.05,0.09)	1	0.23 (0.18,0.28)	1	0.28 (0.25,0.31)	1	0.22 (0.2,0.25)	1	0.61 (0.55,0.68)	1
Respondent edu. Level	0.17 (-0.09,0.43)	0.903	0.18 (-0.07,0.42)	0.924	0.39 (0.25,0.53)	1	-0.12 (-0.28,0.03)	0.06	0.34 (0.18,0.5)	1
Wealth Index†	0.11 (-0.04,0.27)	0.922	-0.08 (-0.21,0.05)	0.11	-0.03 (-0.13,0.07)	0.291	0.06 (-0.02,0.14)	0.922	0.05 (-0.08,0.18)	0.778
**Level 2a (Interviewer)**										
Interviewer age‡	0.04 (0,0.09)	0.968	0.04 (-0.03,0.13)	0.874	-0.02 (-0.1,0.07)	0.328	-0.01 (-0.03,0.02)	0.346	0.01 (-0.05,0.07)	0.65
Interviewer survey experience	-0.24 (-0.85,0.35)	0.209	0.3 (-1.33,1.9)	0.651	0.06 (-1.28,1.41)	0.536	-0.55 (-1.05,-0.05)	0.018	0.08 (-0.64,0.8)	0.587
**Level 2b (Survey cluster)**										
Rural	-0.35 (-0.76,0.06)	0.045	0.29 (-0.09,0.66)	0.936	0.18 (-0.06,0.41)	0.93	-0.4 (-0.64,-0.17)	< 0.001	0.13 (-0.24,0.5)	0.754
**Variance Components**										
Cluster-specific variance	0.12 (0 , 0.58)		0.471 (0.134 , 0.958)		0.329 (0.103 , 0.62)		0.022 (0 , 0.128)		0.267 (0.059 , 0.555)	
Interviewer-specific variance	0.568 (0.222 , 1.217)		0.447 (0.137 , 1.202)		4.657 (3.157 , 6.892)		0.182 (0.069 , 0.44)		0.665 (0.381 , 1.126)	
# of clusters	415		362		1283		485		546	
# of fieldworkers	50		19		74		25		50	
# of observations	1693		1341		5105		2857		2906	

* Respondent age in years centered at 15
† Wealth index centered at 2 of 5, with 5 being highest relative wealth
‡ Interviewer age in years centered at 18

Table S6. Log odds, 95% credible intervals, and posterior probability that log odds are greater than 0 from full model of ever reporting sexual intercourse among never-unioned men (Continued)

	**Timor-Leste (2016)**		**Uganda (2016)**		**South Africa (2016)**		**Zambia (2018)**		**Zimbabwe (2015)**	
	Estimate (95% CrI)	Posterior Prob. > 0	Estimate (95% CrI)	Posterior Prob. > 0	Estimate (95% CrI)	Posterior Prob. > 0	Estimate (95% CrI)	Posterior Prob. > 0	Estimate (95% CrI)	Posterior Prob. > 0
Intercept	-4.39 (-6.09,-2.75)		-1.68 (-2.53,-0.85)		-2.91 (-4.02,-1.84)		0.37 (-1.44,2.18)		-1.35 (-2,-0.72)	
**Level 1 (Respondent)**										
Age difference ≥10 years	-1.28 (-1.79, -0.79)	0	-0.75 (-1.13, -0.38)	< 0.001	-1.53 (-2.02, -1.06)	0	1.14 (0.48, 1.77)	> 0.999	-0.7 (-0.96, -0.44)	0
Respondent age*	0.15 (0.13,0.18)	1	0.22 (0.18,0.26)	1	0.18 (0.15,0.22)	1	0.35 (0.32,0.37)	1	0.24 (0.21,0.27)	1
Respondent edu. Level	0.36 (0.16,0.56)	> 0.999	0.34 (0.16,0.53)	> 0.999	1.15 (0.82,1.49)	1	0.31 (0.16,0.45)	> 0.999	-0.24 (-0.42,-0.05)	0.006
Wealth Index†	0.05 (-0.1,0.2)	0.738	0.1 (0,0.2)	0.976	-0.22 (-0.37,-0.08)	< 0.001	-0.09 (-0.17,-0.01)	0.015	0.05 (-0.05,0.15)	0.847
**Level 2a (Interviewer)**										
Interviewer age‡	0.06 (-0.06,0.18)	0.847	0.02 (-0.02,0.06)	0.809	0.05 (0.02,0.1)	0.995	-0.03 (-0.06,0)	0.036	0.03 (0,0.06)	0.965
Interviewer survey experience	0.18 (-1.2,1.58)	0.606	-0.34 (-0.93,0.26)	0.129	0.06 (-0.74,0.87)	0.565	-0.82 (-2.6,0.96)	0.181	-0.39 (-0.72,-0.07)	0.008
**Level 2b (Survey cluster)**										
Rural	-0.77 (-1.25,-0.31)	< 0.001	0.2 (-0.1,0.51)	0.902	-0.18 (-0.59,0.23)	0.194	0.42 (0.2,0.64)	> 0.999	0.06 (-0.24,0.36)	0.649
**Variance Components**										
Cluster-specific variance	0.609 (0.211,1.212)		0.188 (0.007,0.482)		0.438 (0.09,0.938)		0.195 (0.085,0.334)		0.192 (0.073,0.347)	
Interviewer-specific variance	2.331 (1.165,4.57)		0.383 (0.188,0.732)		0.519 (0.216,1.119)		0.597 (0.347,1.027)		0.132 (0.058,0.261)	
# of clusters	387		628		626		544		394	
# of fieldworkers	32		39		30		39		60	
# of observations	1712		2029		2241		4934		3265	

* Respondent age in years centered at 15
† Wealth index centered at 2 of 5, with 5 being highest relative wealth
‡ Interviewer age in years centered at 18

Sensitivity analysis models using half-Cauchy priors

Table S7. Log odds, 95% credible intervals, and posterior probability that log odds are greater than 0 from sensitivity model of ever reporting sexual intercourse among never-unioned women, using half-Cauchy priors

	Benin (2017-18)		Burundi (2016-17)		Cameroon (2018)		Ethiopia (2016)		Gambia (2019-20)	
	Estimate (95% CrI)	Posterior Prob. > 0	Estimate (95% CrI)	Posterior Prob. > 0	Estimate (95% CrI)	Posterior Prob. > 0	Estimate (95% CrI)	Posterior Prob. > 0	Estimate (95% CrI)	Posterior Prob. > 0
Intercept	-1.42 (-1.93,-0.91)		-1.64 (-2.4,-0.89)		-1.88 (-2.74,-1.03)		-3.29 (-4.07,-2.53)		-2.52 (-3.13,-1.94)	
**Level 1 (Respondent)**										
Age difference ≥10 years	-0.42 (-0.73,-0.12)	0.003	-0.26 (-0.52,0.01)	0.028	-0.44 (-0.75,-0.13)	0.002	-0.49 (-1.05,0.03)	0.032	-0.27 (-0.76,0.21)	0.136
Respondent age*	0.42 (0.38,0.46)	1	0.11 (0.09,0.13)	1	0.43 (0.39,0.46)	1	0.17 (0.15,0.19)	1	0.25 (0.22,0.29)	1
Respondent edu. Level	0.04 (-0.06,0.14)	0.78	-0.07 (-0.18,0.04)	0.107	0.27 (0.11,0.44)	> 0.999	0.2 (0.07,0.34)	0.999	-0.11 (-0.28,0.06)	0.104
Wealth Index†	0.02 (-0.05,0.1)	0.708	0.05 (-0.02,0.13)	0.912	-0.17 (-0.27,-0.06)	< 0.001	-0.04 (-0.19,0.12)	0.319	-0.32 (-0.46,-0.19)	0
**Level 2a (Interviewer)**										
Interviewer age‡	0 (-0.04,0.04)	0.478	0.01 (-0.02,0.03)	0.68	0.04 (-0.01,0.1)	0.936	0.01 (-0.06,0.08)	0.593	0 (-0.04,0.04)	0.507
Interviewer survey experience	0.12 (-0.3,0.53)	0.72	-0.27 (-0.81,0.29)	0.173	-0.48 (-1.12,0.15)	0.065	0.05 (-0.37,0.46)	0.591	0.03 (-0.38,0.44)	0.555
**Level 2b (Survey cluster)**										
Rural	0.05 (-0.16,0.26)	0.676	-0.69 (-0.94,-0.46)	0	0.21 (-0.08,0.5)	0.92	-0.89 (-1.36,-0.43)	0	-0.72 (-1.18,-0.27)	< 0.001
**Variance Components**										
Cluster-specific variance	0.26 (0.126, 0.435)		0.249 (0.129, 0.404)		0.475 (0.302, 0.697)		0.692 (0.378, 1.123)		0.459 (0.195, 0.827)	
Interviewer-specific variance	0.504 (0.313, 0.791)		0.221 (0.118, 0.381)		1.257 (0.805, 1.938)		0.537 (0.256, 0.951)		0.075 (0.001, 0.267)	
# of clusters	545		554		428		619		280	
# of fieldworkers	73		75		68		131		51	
# of observations	3897		6091		4915		4250		3226	

* Respondent age in years centered at 15
† Wealth index centered at 2 of 5, with 5 being highest relative wealth
‡ Interviewer age in years centered at 18

Table S7. Log odds, 95% credible intervals, and posterior probability that log odds are greater than 0 from sensitivity model of ever reporting sexual intercourse among never-unioned women, using half-Cauchy priors. (continued)

	Guinea (2018)		Haiti (2017-18)		Liberia (2019-20)		Mali (2018)		Malawi (2015-16)	
	Estimate (95% CrI)	Posterior Prob. > 0	Estimate (95% CrI)	Posterior Prob. > 0	Estimate (95% CrI)	Posterior Prob. > 0	Estimate (95% CrI)	Posterior Prob. > 0	Estimate (95% CrI)	Posterior Prob. > 0
Intercept	-2.01 (-3.13,-0.91)		-0.78 (-1.2,-0.38)		1 (0.22,1.78)		-2.68 (-3.67,-1.7)		-1.12 (-1.48,-0.77)	
**Level 1 (Respondent)**										
Age difference ≥10 years	-0.28 (-0.65,0.1)	0.072	-0.07 (-0.28,0.14)	0.249	-0.61 (-1.14,-0.05)	0.018	-0.07 (-0.53,0.39)	0.379	-0.28 (-0.65,0.08)	0.061
Respondent age*	0.35 (0.3,0.39)	1	0.21 (0.19,0.23)	1	0.66 (0.58,0.75)	1	0.27 (0.23,0.32)	1	0.31 (0.29,0.35)	1
Respondent edu. Level	-0.08 (-0.2,0.05)	0.109	0.32 (0.2,0.44)	1	0.33 (0.1,0.57)	0.997	0 (-0.16,0.15)	0.483	-0.09 (-0.23,0.05)	0.113
Wealth Index†	-0.19 (-0.32,-0.05)	0.004	-0.11 (-0.19,-0.05)	< 0.001	-0.38 (-0.52,-0.25)	0	0.12 (-0.03,0.27)	0.944	-0.03 (-0.09,0.03)	0.15
**Level 2a (Interviewer)**										
Interviewer age‡	0.03 (-0.04,0.1)	0.823	-0.01 (-0.03,0)	0.027	-0.01 (-0.05,0.03)	0.285	0.02 (-0.03,0.08)	0.781	0.03 (-0.01,0.06)	0.939
Interviewer survey experience	0.12 (-0.69,0.9)	0.614	0.18 (-0.14,0.5)	0.867	-0.44 (-0.9,0.02)	0.03	0.3 (-0.49,1.09)	0.772	0.09 (-0.19,0.37)	0.735
**Level 2b (Survey cluster)**										
Rural	-0.15 (-0.52,0.23)	0.214	-0.42 (-0.63,-0.22)	0	0.05 (-0.33,0.44)	0.606	-0.04 (-0.53,0.45)	0.434	-0.12 (-0.32,0.07)	0.11
**Variance Components**										
Cluster-specific variance	0.209 (0.039, 0.458)		0.24 (0.153, 0.35)		0.366 (0.12, 0.717)		0.539 (0.249, 0.955)		0.169 (0.06, 0.306)	
Interviewer-specific variance	1.6 (0.983, 2.563)		0.023 (0, 0.072)		0.197 (0.033, 0.486)		0.937 (0.503, 1.624)		0.357 (0.236, 0.522)	
# of clusters	376		450		319		307		810	
# of fieldworkers	61		55		54		71		140	
# of observations	2645		5760		2621		1816		4993	

* Respondent age in years centered at 15
† Wealth index centered at 2 of 5, with 5 being highest relative wealth
‡ Interviewer age in years centered at 18

Table S7. Log odds, 95% credible intervals, and posterior probability that log odds are greater than 0 from sensitivity model of ever reporting sexual intercourse among never-unioned women, using half-Cauchy priors (continued)

	**Nigeria (2018)**		**Philippines (2017)**		**Rwanda (2019-20)**		**Sierra Leone (2019)**		**Timor-Leste (2016)**	
	Estimate (95% CrI)	Posterior Prob. > 0	Estimate (95% CrI)	Posterior Prob. > 0	Estimate (95% CrI)	Posterior Prob. > 0	Estimate (95% CrI)	Posterior Prob. > 0	Estimate (95% CrI)	Posterior Prob. > 0
Intercept	-3 (-4.3,-1.71)		-2.74 (-3.25,-2.24)		-0.86 (-1.18,-0.54)		-0.24 (-1,0.53)		-5.05 (-6.32,-3.9)	
**Level 1 (Respondent)**										
Age difference ≥10 years	0.35 (0.1,0.61)	0.997	-0.09 (-0.34,0.15)	0.228	-0.25 (-0.46,-0.04)	0.011	-0.33 (-0.67,0.02)	0.032	-1.41 (-2.1,-0.76)	0
Respondent age*	0.34 (0.32,0.36)	1	0.12 (0.11,0.13)	1	0.17 (0.15,0.18)	1	0.68 (0.63,0.73)	1	0.13 (0.1,0.15)	1
Respondent edu. Level	0.07 (-0.03,0.17)	0.926	0.34 (0.22,0.46)	1	-0.36 (-0.47,-0.25)	0	0.22 (0.09,0.35)	> 0.999	0.03 (-0.22,0.27)	0.583
Wealth Index†	-0.2 (-0.27,-0.14)	0	-0.15 (-0.21,-0.08)	0	-0.03 (-0.09,0.03)	0.144	-0.24 (-0.34,-0.14)	0	-0.15 (-0.33,0.03)	0.054
**Level 2a (Interviewer)**										
Interviewer age‡	0.01 (-0.05,0.06)	0.594	-0.02 (-0.03,0)	0.007	0.01 (-0.01,0.02)	0.889	-0.02 (-0.07,0.02)	0.148	0.08 (0,0.16)	0.981
Interviewer survey experience	-0.02 (-1.05,1)	0.484	-0.08 (-0.47,0.31)	0.35	-0.04 (-0.2,0.13)	0.339	-0.21 (-0.71,0.28)	0.193	-0.42 (-1.1,0.27)	0.112
**Level 2b (Survey cluster)**										
Rural	0.1 (-0.06,0.27)	0.891	-0.52 (-0.71,-0.32)	0	-0.39 (-0.57,-0.22)	0	0.08 (-0.21,0.37)	0.695	-0.18 (-0.78,0.4)	0.269
**Variance Components**										
Cluster-specific variance	0.316 (0.219, 0.432)		0.305 (0.159, 0.485)		0.105 (0.031, 0.2)		0.339 (0.186, 0.535)		0.987 (0.311, 2.058)	
Interviewer-specific variance	3.389 (2.37, 4.854)		0.454 (0.312, 0.64)		0.012 (0, 0.054)		0.457 (0.266, 0.758)		1.192 (0.606, 2.187)	
# of clusters	1328		1230		500		557		452	
# of fieldworkers	116		262		56		66		75	
# of observations	10669		8652		5961		4966		4412	

* Respondent age in years centered at 15
† Wealth index centered at 2 of 5, with 5 being highest relative wealth
‡ Interviewer age in years centered at 18

Table S7. Log odds, 95% credible intervals, and posterior probability that log odds are greater than 0 from sensitivity model of ever reporting sexual intercourse among never-unioned women, using half-Cauchy priors (continued)

	**Uganda (2016)**		**South Africa (2016)**		**Zambia (2018)**		**Zimbabwe (2015)**	
	Estimate (95% CrI)	Posterior Prob. > 0	Estimate (95% CrI)	Posterior Prob. > 0	Estimate (95% CrI)	Posterior Prob. > 0	Estimate (95% CrI)	Posterior Prob. > 0
Intercept	-1.26 (-1.68,-0.84)		-0.34 (-1.08,0.4)		-0.61 (-1.16,-0.06)		-1.44 (-2.4,-0.52)	
**Level 1 (Respondent)**								
Age difference ≥10 years	-0.37 (-0.6,-0.14)	< 0.001	-0.32 (-0.63,0)	0.023	0.14 (-0.18,0.47)	0.807	-0.92 (-1.32,-0.53)	0
Respondent age*	0.26 (0.23,0.29)	1	0.36 (0.33,0.4)	1	0.4 (0.36,0.43)	1	0.21 (0.18,0.24)	1
Respondent edu. Level	0.22 (0.09,0.34)	> 0.999	0.26 (0,0.53)	0.974	0.07 (-0.09,0.23)	0.789	-0.27 (-0.53,-0.01)	0.019
Wealth Index†	0.01 (-0.06,0.08)	0.626	-0.35 (-0.44,-0.26)	0	-0.26 (-0.35,-0.17)	0	-0.14 (-0.29,0.01)	0.035
**Level 2a (Interviewer)**								
Interviewer age‡	0 (-0.03,0.03)	0.499	-0.01 (-0.04,0.01)	0.149	-0.01 (-0.03,0.01)	0.207	0.08 (0.02,0.15)	0.993
Interviewer survey experience	-0.23 (-0.52,0.06)	0.059	0.14 (-0.26,0.55)	0.761	-0.03 (-0.41,0.34)	0.429	-0.42 (-0.89,0.08)	0.048
**Level 2b (Survey cluster)**								
Rural	0.08 (-0.12,0.28)	0.775	-0.17 (-0.42,0.09)	0.101	0.1 (-0.14,0.34)	0.79	-0.05 (-0.5,0.39)	0.413
**Variance Components**								
Cluster-specific variance	0.152 (0.054, 0.282)		0.075 (0.001, 0.256)		0.255 (0.126, 0.422)		0.574 (0.299, 0.949)	
Interviewer-specific variance	0.226 (0.131, 0.365)		0.257 (0.14, 0.43)		0.345 (0.203, 0.562)		0.427 (0.211, 0.787)	
# of clusters	685		687		537		391	
# of fieldworkers	86		91		66		58	
# of observations	4567		5134		4105		2386	

* Respondent age in years centered at 15
† Wealth index centered at 2 of 5, with 5 being highest relative wealth
‡ Interviewer age in years centered at 18

Table S8. Log odds, 95% credible intervals, and posterior probability that log odds are greater than 0 from sensitivity model of ever reporting sexual intercourse among never-unioned men, using half-Cauchy priors

	Benin (2017-18)		Burundi (2016-17)		Cameroon (2018)		Ethiopia (2016)		Gambia (2019-20)	
	Estimate (95% CrI)	Posterior Prob. > 0	Estimate (95% CrI)	Posterior Prob. > 0	Estimate (95% CrI)	Posterior Prob. > 0	Estimate (95% CrI)	Posterior Prob. > 0	Estimate (95% CrI)	Posterior Prob. > 0
Intercept	-1.95 (-2.94,-0.99)		-2.54 (-4.88,-0.32)		-2.9 (-4.52,-1.29)		-1.99 (-3.13,-0.86)		-1.57 (-2.22,-0.91)	
**Level 1 (Respondent)**										
Age difference ≥10 years	0.42 (0.03,0.82)	0.982	0.7 (0.24,1.15)	0.999	0.51 (0.12,0.9)	0.996	-0.66 (-0.92,-0.39)	0	-0.25 (-0.59,0.09)	0.075
Respondent age*	0.52 (0.47,0.58)	1	0.18 (0.15,0.2)	1	0.38 (0.34,0.42)	1	0.17 (0.14,0.19)	1	0.19 (0.16,0.22)	1
Respondent edu. Level	0.24 (0.11,0.38)	> 0.999	-0.28 (-0.43,-0.14)	< 0.001	0.38 (0.22,0.55)	1	0.3 (0.19,0.42)	1	0.15 (0.03,0.27)	0.995
Wealth Index†	0 (-0.1,0.09)	0.469	0.05 (-0.05,0.15)	0.847	0.15 (0.03,0.27)	0.994	0.09 (-0.01,0.18)	0.967	-0.01 (-0.11,0.09)	0.401
**Level 2a (Interviewer)**										
Interviewer age‡	-0.07 (-0.13,0)	0.027	0.01 (-0.06,0.08)	0.596	-0.02 (-0.12,0.08)	0.337	0.01 (-0.04,0.05)	0.611	0.02 (-0.02,0.05)	0.809
Interviewer survey experience	-0.34 (-1.11,0.43)	0.19	0.14 (-1.92,2.16)	0.552	-0.3 (-1.3,0.71)	0.279	-0.54 (-1.58,0.47)	0.142	0.13 (-0.4,0.68)	0.695
**Level 2b (Survey cluster)**										
Rural	0.04 (-0.22,0.3)	0.618	-0.45 (-0.76,-0.16)	0.001	0.27 (-0.02,0.55)	0.967	-0.4 (-0.75,-0.07)	0.009	-0.31 (-0.64,0.01)	0.03
**Variance Components**										
Cluster-specific variance	0.375 (0.154, 0.672)		0.281 (0.114, 0.51)		0.194 (0.037, 0.409)		0.587 (0.375, 0.857)		0.204 (0.067, 0.393)	
Interviewer-specific variance	0.79 (0.409, 1.484)		0.935 (0.451, 1.909)		1.332 (0.759, 2.362)		1.214 (0.749, 1.951)		0.132 (0.037, 0.356)	
# of clusters	545		499		427		619		279	
# of fieldworkers	37		31		34		58		22	
# of observations	2906		2825		3380		4600		2384	

* Respondent age in years centered at 15
† Wealth index centered at 2 of 5, with 5 being highest relative wealth
‡ Interviewer age in years centered at 18

Table S8. Log odds, 95% credible intervals, and posterior probability that log odds are greater than 0 from sensitivity model of ever reporting sexual intercourse among never-unioned men, using half-Cauchy priors (Continued)

	Guinea (2018)		Haiti (2017-18)		Liberia (2019-20)		Mali (2018)		Malawi (2015-16)	
	Estimate (95% CrI)	Posterior Prob. > 0	Estimate (95% CrI)	Posterior Prob. > 0	Estimate (95% CrI)	Posterior Prob. > 0	Estimate (95% CrI)	Posterior Prob. > 0	Estimate (95% CrI)	Posterior Prob. > 0
Intercept	-2.97 (-4.72,-1.29)		-1.39 (-1.97,-0.82)		-4.02 (-5.73,-2.36)		-3.84 (-5.8,-1.91)		0.24 (-0.29,0.77)	
**Level 1 (Respondent)**										
Age difference ≥10 years	0.9 (0.25,1.54)	0.997	0.05 (-0.32,0.42)	0.6	2.32 (1.3,3.32)	1	1.12 (0.52,1.73)	> 0.999	-0.54 (-0.83,-0.24)	< 0.001
Respondent age*	0.33 (0.29,0.38)	1	0.27 (0.24,0.3)	1	0.48 (0.42,0.55)	1	0.44 (0.38,0.5)	1	0.22 (0.18,0.26)	1
Respondent edu. Level	0.21 (0.05,0.36)	0.996	0.9 (0.74,1.05)	1	0.9 (0.64,1.16)	1	0.31 (0.14,0.48)	> 0.999	0.12 (-0.08,0.31)	0.882
Wealth Index†	0.02 (-0.12,0.17)	0.611	0.09 (0,0.17)	0.978	-0.05 (-0.19,0.09)	0.245	0.07 (-0.1,0.24)	0.789	-0.08 (-0.16,0)	0.027
**Level 2a (Interviewer)**										
Interviewer age‡	0.02 (-0.05,0.09)	0.723	-0.01 (-0.03,0.01)	0.107	-0.01 (-0.07,0.05)	0.308	-0.05 (-0.18,0.08)	0.231	0 (-0.02,0.02)	0.396
Interviewer survey experience	-0.44 (-1.95,1.05)	0.277	-0.01 (-0.38,0.36)	0.48	-0.02 (-0.77,0.71)	0.482	0.56 (-0.78,1.94)	0.799	-0.08 (-0.48,0.31)	0.337
**Level 2b (Survey cluster)**										
Rural	0.04 (-0.37,0.45)	0.57	-0.27 (-0.51,-0.03)	0.013	-0.32 (-0.71,0.07)	0.055	-0.41 (-0.94,0.13)	0.065	-0.01 (-0.27,0.25)	0.46
**Variance Components**										
Cluster-specific variance	0.139 (0.002, 0.454)		0.127 (0.021, 0.27)		0.325 (0.042, 0.751)		0.301 (0.013, 0.789)		0.075 (0, 0.279)	
Interviewer-specific variance	2.361 (1.341, 4.139)		0.092 (0.027, 0.218)		0.361 (0.121, 0.872)		2.626 (1.446, 4.686)		0.309 (0.159, 0.553)	
# of clusters	363		450		311		322		757	
# of fieldworkers	39		30		29		45		67	
# of observations	1761		4608		1543		1580		2666	

* Respondent age in years centered at 15
† Wealth index centered at 2 of 5, with 5 being highest relative wealth
‡ Interviewer age in years centered at 18

Table S8. Log odds, 95% credible intervals, and posterior probability that log odds are greater than 0 from sensitivity model of ever reporting sexual intercourse among never-unioned men, using half-Cauchy priors (Continued)

	**Myanmar (2015-16)**		**Nepal (2016)**		**Nigeria (2018)**		**Rwanda (2019-20)**		**Sierra Leone (2019)**	
	Estimate (95% CrI)	Posterior Prob. > 0	Estimate (95% CrI)	Posterior Prob. > 0	Estimate (95% CrI)	Posterior Prob. > 0	Estimate (95% CrI)	Posterior Prob. > 0	Estimate (95% CrI)	Posterior Prob. > 0
Intercept	-2.65 (-3.52,-1.82)		-3.11 (-4.9,-1.37)		-4.23 (-6.03,-2.41)		-1.02 (-1.78,-0.26)		-2.96 (-4.34,-1.62)	
**Level 1 (Respondent)**										
Age difference ≥10 years	-1.48 (-2.06,-0.95)	0	0.41 (-0.13,0.95)	0.933	0.82 (0.42,1.21)	1	0.25 (-0.14,0.63)	0.893	0.35 (-0.4,1.04)	0.822
Respondent age*	0.04 (0.02,0.07)	> 0.999	0.29 (0.23,0.35)	1	0.3 (0.28,0.33)	1	0.24 (0.21,0.27)	1	0.63 (0.57,0.69)	1
Respondent edu. Level	0.15 (-0.1,0.42)	0.88	0.19 (-0.05,0.44)	0.943	0.38 (0.24,0.52)	1	-0.12 (-0.28,0.04)	0.063	0.34 (0.18,0.5)	1
Wealth Index†	0.1 (-0.06,0.26)	0.881	-0.08 (-0.21,0.05)	0.105	-0.03 (-0.13,0.07)	0.302	0.06 (-0.02,0.14)	0.92	0.05 (-0.08,0.18)	0.783
**Level 2a (Interviewer)**										
Interviewer age‡	0.05 (0.01,0.09)	0.993	0 (-0.08,0.07)	0.455	-0.03 (-0.11,0.05)	0.226	-0.02 (-0.04,0.01)	0.095	0 (-0.05,0.06)	0.571
Interviewer survey experience	-0.18 (-0.79,0.44)	0.276	0.24 (-1.36,1.79)	0.624	0.02 (-1.34,1.38)	0.514	-0.52 (-1.01,-0.03)	0.02	0.1 (-0.63,0.82)	0.607
**Level 2b (Survey cluster)**										
Rural	-0.39 (-0.8,0.03)	0.033	0.29 (-0.08,0.66)	0.94	0.18 (-0.06,0.41)	0.93	-0.4 (-0.64,-0.17)	< 0.001	0.13 (-0.24,0.49)	0.754
**Variance Components**										
Cluster-specific variance	0.128 (0, 0.599)		0.416 (0.099, 0.891)		0.316 (0.095, 0.601)		0.022 (0, 0.13)		0.274 (0.066, 0.566)	
Interviewer-specific variance	0.627 (0.256, 1.316)		0.414 (0.12, 1.134)		4.575 (3.113, 6.74)		0.176 (0.066, 0.432)		0.671 (0.385, 1.132)	
# of clusters	415		362		1283		485		546	
# of fieldworkers	50		19		74		25		50	
# of observations	1693		1341		5105		2857		2906	

* Respondent age in years centered at 15
† Wealth index centered at 2 of 5, with 5 being highest relative wealth
‡ Interviewer age in years centered at 18

Table S8. Log odds, 95% credible intervals, and posterior probability that log odds are greater than 0 from sensitivity model of ever reporting sexual intercourse among never-unioned men, using half-Cauchy priors (Continued)

	**Timor-Leste (2016)**		**Uganda (2016)**		**South Africa (2016)**		**Zambia (2018)**		**Zimbabwe (2015)**	
	Estimate (95% CrI)	Posterior Prob. > 0	Estimate (95% CrI)	Posterior Prob. > 0	Estimate (95% CrI)	Posterior Prob. > 0	Estimate (95% CrI)	Posterior Prob. > 0	Estimate (95% CrI)	Posterior Prob. > 0
Intercept	-2.74 (-4.44,-1.11)		-1.71 (-2.57,-0.86)		-1.64 (-2.67,-0.64)		-3.06 (-5.14,-0.89)		-0.83 (-1.5,-0.18)	
**Level 1 (Respondent)**										
Age difference ≥10 years	-0.64 (-1.12,-0.16)	0.005	0.66 (0.18,1.14)	0.997	-0.48 (-1,0.03)	0.032	3.38 (2.13,4.43)	1	0.03 (-0.31,0.36)	0.566
Respondent age*	0.16 (0.13,0.2)	1	0.3 (0.25,0.35)	1	0.22 (0.18,0.27)	1	0.35 (0.32,0.38)	1	0.29 (0.26,0.33)	1
Respondent edu. Level	0.4 (0.21,0.61)	> 0.999	0.34 (0.15,0.53)	> 0.999	1.24 (0.92,1.58)	1	0.29 (0.15,0.44)	> 0.999	-0.22 (-0.4,-0.04)	0.008
Wealth Index†	0.03 (-0.12,0.17)	0.647	0.1 (0,0.2)	0.979	-0.22 (-0.36,-0.09)	< 0.001	-0.09 (-0.17,-0.01)	0.018	0.06 (-0.04,0.16)	0.871
**Level 2a (Interviewer)**										
Interviewer age‡	0.01 (-0.11,0.13)	0.597	-0.03 (-0.06,0.01)	0.078	0.02 (-0.01,0.05)	0.895	-0.03 (-0.06,0.01)	0.048	-0.01 (-0.04,0.02)	0.231
Interviewer survey experience	0.19 (-1.19,1.61)	0.614	-0.34 (-0.89,0.2)	0.102	-0.1 (-0.75,0.54)	0.373	-0.82 (-2.6,0.96)	0.176	-0.39 (-0.7,-0.09)	0.005
**Level 2b (Survey cluster)**										
Rural	-0.77 (-1.24,-0.31)	< 0.001	0.18 (-0.12,0.49)	0.884	-0.12 (-0.51,0.27)	0.275	0.43 (0.21,0.65)	> 0.999	0.05 (-0.25,0.34)	0.618
**Variance Components**										
Cluster-specific variance	0.51 (0.147, 1.073)		0.183 (0.009, 0.465)		0.321 (0.026, 0.762)		0.198 (0.09, 0.336)		0.185 (0.069, 0.338)	
Interviewer-specific variance	2.437 (1.226, 4.831)		0.31 (0.147, 0.599)		0.282 (0.095, 0.647)		0.592 (0.344, 1.018)		0.114 (0.048, 0.232)	
# of clusters	387		628		626		544		394	
# of fieldworkers	32		39		30		39		60	
# of observations	1712		2029		2241		4934		3265	

* Respondent age in years centered at 15
† Wealth index centered at 2 of 5, with 5 being highest relative wealth
‡ Interviewer age in years centered at 18

Sensitivity analysis models using age difference of five or more years

Table S9. Log odds, 95% credible intervals, and posterior probability that log odds are greater than 0 from sensitivity model of ever reporting sexual intercourse among never-unioned women, using age difference of five or more years

	Benin (2017-18)		Burundi (2016-17)		Cameroon (2018)		Ethiopia (2016)		Gambia (2019-20)	
	Estimate (95% CrI)	Posterior Prob. > 0	Estimate (95% CrI)	Posterior Prob. > 0	Estimate (95% CrI)	Posterior Prob. > 0	Estimate (95% CrI)	Posterior Prob. > 0	Estimate (95% CrI)	Posterior Prob. > 0
Intercept	-1.57 (-2.13,-1.02)		-2.69 (-3.54,-1.88)		-2.07 (-2.96,-1.17)		-2.76 (-3.56,-1.99)		-2.3 (-2.94,-1.68)	
**Level 1 (Respondent)**										
Age difference ≥5 years	0.2 (-0.13,0.52)	0.884	1.03 (0.67,1.4)	1	0.18 (-0.19,0.55)	0.833	-0.92 (-1.25,-0.58)	0	-0.48 (-0.91,-0.05)	0.015
Respondent age*	0.46 (0.42,0.5)	1	0.16 (0.14,0.18)	1	0.46 (0.43,0.5)	1	0.13 (0.11,0.16)	1	0.24 (0.21,0.28)	1
Respondent edu. Level	0.04 (-0.06,0.15)	0.784	-0.1 (-0.21,0.02)	0.045	0.27 (0.11,0.44)	> 0.999	0.15 (0.02,0.29)	0.987	-0.12 (-0.28,0.05)	0.086
Wealth Index†	0.02 (-0.06,0.09)	0.696	0.05 (-0.03,0.12)	0.897	-0.17 (-0.27,-0.06)	< 0.001	-0.04 (-0.19,0.11)	0.302	-0.33 (-0.47,-0.2)	0
**Level 2a (Interviewer)**										
Interviewer age‡	-0.03 (-0.07,0)	0.035	-0.01 (-0.04,0.01)	0.15	0.01 (-0.04,0.07)	0.7	0.03 (-0.04,0.09)	0.811	0 (-0.03,0.04)	0.608
Interviewer survey experience	0.05 (-0.35,0.46)	0.6	-0.24 (-0.8,0.34)	0.204	-0.49 (-1.12,0.12)	0.058	0.11 (-0.32,0.55)	0.691	0.07 (-0.33,0.49)	0.638
**Level 2b (Survey cluster)**										
Rural	0.05 (-0.16,0.26)	0.674	-0.7 (-0.95,-0.46)	0	0.2 (-0.09,0.49)	0.912	-0.89 (-1.36,-0.43)	< 0.001	-0.73 (-1.2,-0.28)	< 0.001
**Variance Components**										
Cluster-specific variance	0.259 (0.125, 0.432)		0.254 (0.133, 0.409)		0.474 (0.302, 0.696)		0.708 (0.386, 1.144)		0.5 (0.22, 0.887)	
Interviewer-specific variance	0.477 (0.294, 0.755)		0.249 (0.138, 0.422)		1.239 (0.791, 1.921)		0.611 (0.309, 1.058)		0.073 (0.001, 0.267)	
# of clusters	545		554		428		619		280	
# of fieldworkers	73		75		68		131		51	
# of observations	3897		6091		4915		4250		3226	

* Respondent age in years centered at 15
† Wealth index centered at 2 of 5, with 5 being highest relative wealth
‡ Interviewer age in years centered at 18

Table S9. Log odds, 95% credible intervals, and posterior probability that log odds are greater than 0 from sensitivity model of ever reporting sexual intercourse among never-unioned women, using age difference of five or more years (continued)

	**Guinea (2018)**		**Haiti (2017-18)**		**Liberia (2019-20)**		**Mali (2018)**		**Malawi (2015-16)**	
	Estimate (95% CrI)	Posterior Prob. > 0	Estimate (95% CrI)	Posterior Prob. > 0	Estimate (95% CrI)	Posterior Prob. > 0	Estimate (95% CrI)	Posterior Prob. > 0	Estimate (95% CrI)	Posterior Prob. > 0
Intercept	-3.44 (-4.68,-2.24)		-1.17 (-1.63,-0.72)		0.57 (-0.61,1.75)		-2.96 (-4,-1.9)		-0.76 (-1.14,-0.38)	
**Level 1 (Respondent)**										
Age difference ≥5 years	1.59 (1.06,2.12)	1	0.52 (0.24,0.8)	> 0.999	0.24 (-0.7,1.14)	0.7	0.41 (-0.15,0.97)	0.923	-0.55 (-0.77,-0.34)	0
Respondent age*	0.45 (0.4,0.5)	1	0.24 (0.22,0.26)	1	0.7 (0.62,0.79)	1	0.3 (0.25,0.35)	1	0.27 (0.24,0.31)	1
Respondent edu. Level	-0.07 (-0.2,0.06)	0.146	0.32 (0.2,0.44)	1	0.33 (0.1,0.57)	0.997	0 (-0.16,0.15)	0.483	-0.11 (-0.25,0.03)	0.066
Wealth Index†	-0.2 (-0.34,-0.06)	0.003	-0.12 (-0.19,-0.05)	< 0.001	-0.38 (-0.52,-0.25)	0	0.11 (-0.03,0.27)	0.933	-0.03 (-0.1,0.03)	0.138
**Level 2a (Interviewer)**										
Interviewer age‡	-0.01 (-0.07,0.06)	0.412	-0.02 (-0.03,-0.01)	< 0.001	-0.04 (-0.07,0)	0.016	0.01 (-0.05,0.06)	0.612	0.04 (0.01,0.06)	0.99
Interviewer survey experience	0.04 (-0.78,0.85)	0.539	0.06 (-0.26,0.39)	0.654	-0.42 (-0.93,0.08)	0.049	0.28 (-0.5,1.08)	0.762	0.14 (-0.14,0.43)	0.838
**Level 2b (Survey cluster)**										
Rural	-0.17 (-0.55,0.21)	0.189	-0.42 (-0.63,-0.22)	0	0.08 (-0.3,0.47)	0.661	-0.05 (-0.55,0.44)	0.416	-0.13 (-0.33,0.07)	0.099
**Variance Components**										
Cluster-specific variance	0.269 (0.084, 0.53)		0.242 (0.153, 0.352)		0.391 (0.135, 0.752)		0.57 (0.269, 0.997)		0.166 (0.058, 0.303)	
Interviewer-specific variance	1.641 (1.001, 2.645)		0.028 (0.001, 0.08)		0.301 (0.102, 0.647)		0.946 (0.5, 1.658)		0.362 (0.24, 0.526)	
# of clusters	376		450		319		307		810	
# of fieldworkers	61		55		54		71		140	
# of observations	2645		5760		2621		1816		4993	

* Respondent age in years centered at 15
† Wealth index centered at 2 of 5, with 5 being highest relative wealth
‡ Interviewer age in years centered at 18

Table S9. Log odds, 95% credible intervals, and posterior probability that log odds are greater than 0 from sensitivity model of ever reporting sexual intercourse among never-unioned women, using age difference of five or more years (continued)

	Nigeria (2018)		Philippines (2017)		Rwanda (2019-20)		Sierra Leone (2019)		Timor-Leste (2016)	
	Estimate (95% CrI)	Posterior Prob. > 0	Estimate (95% CrI)	Posterior Prob. > 0	Estimate (95% CrI)	Posterior Prob. > 0	Estimate (95% CrI)	Posterior Prob. > 0	Estimate (95% CrI)	Posterior Prob. > 0
Intercept	-3.65 (-4.99,-2.3)		-2.62 (-3.14,-2.1)		-1.27 (-1.63,-0.9)		-1.55 (-2.57,-0.5)		-4.61 (-5.94,-3.41)	
**Level 1 (Respondent)**										
Age difference ≥5 years	0.98 (0.63,1.32)	1	-0.27 (-0.52,-0.02)	0.016	0.49 (0.25,0.73)	> 0.999	1.26 (0.51,1.94)	> 0.999	-1.01 (-1.58,-0.43)	< 0.001
Respondent age*	0.35 (0.33,0.37)	1	0.12 (0.1,0.13)	1	0.2 (0.18,0.21)	1	0.72 (0.67,0.78)	1	0.12 (0.09,0.15)	1
Respondent edu. Level	0.07 (-0.04,0.17)	0.898	0.34 (0.22,0.47)	1	-0.36 (-0.47,-0.25)	0	0.22 (0.08,0.35)	> 0.999	0.02 (-0.22,0.27)	0.579
Wealth Index†	-0.21 (-0.27,-0.14)	0	-0.15 (-0.21,-0.09)	0	-0.03 (-0.08,0.03)	0.153	-0.24 (-0.35,-0.14)	0	-0.15 (-0.33,0.03)	0.054
**Level 2a (Interviewer)**										
Interviewer age‡	0.01 (-0.05,0.06)	0.588	-0.02 (-0.03,0)	0.015	-0.01 (-0.03,0)	0.033	-0.04 (-0.09,0)	0.028	0.06 (-0.01,0.14)	0.946
Interviewer survey experience	-0.01 (-1.04,1)	0.488	-0.06 (-0.45,0.33)	0.381	-0.05 (-0.22,0.11)	0.264	-0.23 (-0.76,0.28)	0.186	-0.38 (-1.09,0.32)	0.138
**Level 2b (Survey cluster)**										
Rural	0.1 (-0.07,0.26)	0.887	-0.52 (-0.71,-0.33)	0	-0.39 (-0.57,-0.21)	0	0.07 (-0.22,0.37)	0.69	-0.19 (-0.8,0.39)	0.261
**Variance Components**										
Cluster-specific variance	0.322 (0.223, 0.439)		0.309 (0.163, 0.491)		0.099 (0.025, 0.196)		0.36 (0.201, 0.564)		1.054 (0.343, 2.161)	
Interviewer-specific variance	3.465 (2.415, 4.965)		0.46 (0.314, 0.645)		0.013 (0, 0.058)		0.508 (0.296, 0.835)		1.29 (0.657, 2.352)	
# of clusters	1328		1230		500		557		452	
# of fieldworkers	116		262		56		66		75	
# of observations	10669		8652		5961		4966		4412	

* Respondent age in years centered at 15
† Wealth index centered at 2 of 5, with 5 being highest relative wealth
‡ Interviewer age in years centered at 18

Table S9. Log odds, 95% credible intervals, and posterior probability that log odds are greater than 0 from sensitivity model of ever reporting sexual intercourse among never-unioned women, using age difference of five or more years (continued)

	**Uganda (2016)**		**South Africa (2016)**		**Zambia (2018)**		**Zimbabwe (2015)**	
	Estimate (95% CrI)	Posterior Prob. > 0	Estimate (95% CrI)	Posterior Prob. > 0	Estimate (95% CrI)	Posterior Prob. > 0	Estimate (95% CrI)	Posterior Prob. > 0
Intercept	-1.77 (-2.27,-1.26)		-1.18 (-2.01,-0.37)		-0.96 (-1.67,-0.26)		-0.5 (-1.5,0.48)	
**Level 1 (Respondent)**								
Age difference ≥5 years	0.42 (0.11,0.74)	0.996	0.88 (0.46,1.29)	> 0.999	0.48 (-0.05,0.99)	0.96	-1.05 (-1.46,-0.65)	0
Respondent age*	0.31 (0.28,0.34)	1	0.42 (0.38,0.45)	1	0.4 (0.37,0.44)	1	0.18 (0.14,0.21)	1
Respondent edu. Level	0.22 (0.1,0.35)	> 0.999	0.26 (-0.01,0.52)	0.971	0.07 (-0.1,0.23)	0.786	-0.27 (-0.52,-0.01)	0.021
Wealth Index†	0.01 (-0.05,0.08)	0.661	-0.35 (-0.45,-0.26)	0	-0.26 (-0.35,-0.17)	0	-0.13 (-0.29,0.02)	0.039
**Level 2a (Interviewer)**								
Interviewer age‡	-0.02 (-0.05,0)	0.021	-0.04 (-0.06,-0.01)	0.001	-0.01 (-0.03,0.01)	0.209	0.05 (-0.01,0.11)	0.947
Interviewer survey experience	-0.24 (-0.52,0.04)	0.048	0.1 (-0.31,0.52)	0.689	-0.03 (-0.41,0.35)	0.435	-0.45 (-0.93,0.04)	0.034
**Level 2b (Survey cluster)**								
Rural	0.08 (-0.11,0.28)	0.8	-0.15 (-0.41,0.1)	0.123	0.09 (-0.15,0.34)	0.776	-0.05 (-0.5,0.39)	0.411
**Variance Components**								
Cluster-specific variance	0.156 (0.059, 0.282)		0.078 (0.001, 0.264)		0.261 (0.13, 0.428)		0.596 (0.316, 0.979)	
Interviewer-specific variance	0.207 (0.117, 0.341)		0.268 (0.146, 0.445)		0.368 (0.218, 0.6)		0.418 (0.201, 0.782)	
# of clusters	685		687		537		391	
# of fieldworkers	86		91		66		58	
# of observations	4567		5134		4105		2386	

* Respondent age in years centered at 15
† Wealth index centered at 2 of 5, with 5 being highest relative wealth
‡ Interviewer age in years centered at 18

Table S10. Log odds, 95% credible intervals, and posterior probability that log odds are greater than 0 from sensitivity model of ever reporting sexual intercourse among never-unioned men, using age difference of five or more years

	Benin (2017-18)		Burundi (2016-17)		Cameroon (2018)		Ethiopia (2016)		Gambia (2019-20)	
	Estimate (95% CrI)	Posterior Prob. > 0	Estimate (95% CrI)	Posterior Prob. > 0	Estimate (95% CrI)	Posterior Prob. > 0	Estimate (95% CrI)	Posterior Prob. > 0	Estimate (95% CrI)	Posterior Prob. > 0
Intercept	-1.95 (-2.94,-0.99)		-2.54 (-4.88,-0.32)		-2.9 (-4.52,-1.29)		-1.99 (-3.13,-0.86)		-1.57 (-2.22,-0.91)	
**Level 1 (Respondent)**										
Age difference ≥5 years	0.42 (0.03,0.82)	0.982	0.7 (0.24,1.15)	0.999	0.51 (0.12,0.9)	0.996	-0.66 (-0.92,-0.39)	0	-0.25 (-0.59,0.09)	0.075
Respondent age*	0.52 (0.47,0.58)	1	0.18 (0.15,0.2)	1	0.38 (0.34,0.42)	1	0.17 (0.14,0.19)	1	0.19 (0.16,0.22)	1
Respondent edu. Level	0.24 (0.11,0.38)	> 0.999	-0.28 (-0.43,-0.14)	< 0.001	0.38 (0.22,0.55)	1	0.3 (0.19,0.42)	1	0.15 (0.03,0.27)	0.995
Wealth Index†	0 (-0.1,0.09)	0.469	0.05 (-0.05,0.15)	0.847	0.15 (0.03,0.27)	0.994	0.09 (-0.01,0.18)	0.967	-0.01 (-0.11,0.09)	0.401
**Level 2a (Interviewer)**										
Interviewer age‡	-0.07 (-0.13,0)	0.027	0.01 (-0.06,0.08)	0.596	-0.02 (-0.12,0.08)	0.337	0.01 (-0.04,0.05)	0.611	0.02 (-0.02,0.05)	0.809
Interviewer survey experience	-0.34 (-1.11,0.43)	0.19	0.14 (-1.92,2.16)	0.552	-0.3 (-1.3,0.71)	0.279	-0.54 (-1.58,0.47)	0.142	0.13 (-0.4,0.68)	0.695
**Level 2b (Survey cluster)**										
Rural	0.04 (-0.22,0.3)	0.618	-0.45 (-0.76,-0.16)	0.001	0.27 (-0.02,0.55)	0.967	-0.4 (-0.75,-0.07)	0.009	-0.31 (-0.64,0.01)	0.03
**Variance Components**										
Cluster-specific variance	0.375 (0.154, 0.672)		0.281 (0.114, 0.51)		0.194 (0.037, 0.409)		0.587 (0.375, 0.857)		0.204 (0.067, 0.393)	
Interviewer-specific variance	0.79 (0.409, 1.484)		0.935 (0.451, 1.909)		1.332 (0.759, 2.362)		1.214 (0.749, 1.951)		0.132 (0.037, 0.356)	
# of clusters	545		499		427		619		279	
# of fieldworkers	37		31		34		58		22	
# of observations	2906		2825		3380		4600		2384	

* Respondent age in years centered at 15
† Wealth index centered at 2 of 5, with 5 being highest relative wealth
‡ Interviewer age in years centered at 18

Table S10. Log odds, 95% credible intervals, and posterior probability that log odds are greater than 0 from sensitivity model of ever reporting sexual intercourse among never-unioned men, using age difference of five or more years (Continued)

	Guinea (2018)		Haiti (2017-18)		Liberia (2019-20)		Mali (2018)		Malawi (2015-16)	
	Estimate (95% CrI)	Posterior Prob. > 0	Estimate (95% CrI)	Posterior Prob. > 0	Estimate (95% CrI)	Posterior Prob. > 0	Estimate (95% CrI)	Posterior Prob. > 0	Estimate (95% CrI)	Posterior Prob. > 0
Intercept	-2.97 (-4.72,-1.29)		-1.39 (-1.97,-0.82)		-4.02 (-5.73,-2.36)		-3.84 (-5.8,-1.91)		0.24 (-0.29,0.77)	
**Level 1 (Respondent)**										
Age difference ≥5 years	0.9 (0.25,1.54)	0.997	0.05 (-0.32,0.42)	0.6	2.32 (1.3,3.32)	1	1.12 (0.52,1.73)	> 0.999	-0.54 (-0.83,-0.24)	< 0.001
Respondent age*	0.33 (0.29,0.38)	1	0.27 (0.24,0.3)	1	0.48 (0.42,0.55)	1	0.44 (0.38,0.5)	1	0.22 (0.18,0.26)	1
Respondent edu. Level	0.21 (0.05,0.36)	0.996	0.9 (0.74,1.05)	1	0.9 (0.64,1.16)	1	0.31 (0.14,0.48)	> 0.999	0.12 (-0.08,0.31)	0.882
Wealth Index†	0.02 (-0.12,0.17)	0.611	0.09 (0,0.17)	0.978	-0.05 (-0.19,0.09)	0.245	0.07 (-0.1,0.24)	0.789	-0.08 (-0.16,0)	0.027
**Level 2a (Interviewer)**										
Interviewer age‡	0.02 (-0.05,0.09)	0.723	-0.01 (-0.03,0.01)	0.107	-0.01 (-0.07,0.05)	0.308	-0.05 (-0.18,0.08)	0.231	0 (-0.02,0.02)	0.396
Interviewer survey experience	-0.44 (-1.95,1.05)	0.277	-0.01 (-0.38,0.36)	0.48	-0.02 (-0.77,0.71)	0.482	0.56 (-0.78,1.94)	0.799	-0.08 (-0.48,0.31)	0.337
**Level 2b (Survey cluster)**										
Rural	0.04 (-0.37,0.45)	0.57	-0.27 (-0.51,-0.03)	0.013	-0.32 (-0.71,0.07)	0.055	-0.41 (-0.94,0.13)	0.065	-0.01 (-0.27,0.25)	0.46
**Variance Components**										
Cluster-specific variance	0.139 (0.002, 0.454)		0.127 (0.021, 0.27)		0.325 (0.042, 0.751)		0.301 (0.013, 0.789)		0.075 (0, 0.279)	
Interviewer-specific variance	2.361 (1.341, 4.139)		0.092 (0.027, 0.218)		0.361 (0.121, 0.872)		2.626 (1.446, 4.686)		0.309 (0.159, 0.553)	
# of clusters	363		450		311		322		757	
# of fieldworkers	39		30		29		45		67	
# of observations	1761		4608		1543		1580		2666	

* Respondent age in years centered at 15
† Wealth index centered at 2 of 5, with 5 being highest relative wealth
‡ Interviewer age in years centered at 18

Table S10. Log odds, 95% credible intervals, and posterior probability that log odds are greater than 0 from sensitivity model of ever reporting sexual intercourse among never-unioned men, using age difference of five or more years (Continued)

	**Myanmar (2015-16)**		**Nepal (2016)**		**Nigeria (2018)**		**Rwanda (2019-20)**		**Sierra Leone (2019)**	
	Estimate (95% CrI)	Posterior Prob. > 0	Estimate (95% CrI)	Posterior Prob. > 0	Estimate (95% CrI)	Posterior Prob. > 0	Estimate (95% CrI)	Posterior Prob. > 0	Estimate (95% CrI)	Posterior Prob. > 0
Intercept	-2.65 (-3.52,-1.82)		-3.11 (-4.9,-1.37)		-4.23 (-6.03,-2.41)		-1.02 (-1.78,-0.26)		-2.96 (-4.34,-1.62)	
**Level 1 (Respondent)**										
Age difference ≥5 years	-1.48 (-2.06,-0.95)	0	0.41 (-0.13,0.95)	0.933	0.82 (0.42,1.21)	1	0.25 (-0.14,0.63)	0.893	0.35 (-0.4,1.04)	0.822
Respondent age*	0.04 (0.02,0.07)	> 0.999	0.29 (0.23,0.35)	1	0.3 (0.28,0.33)	1	0.24 (0.21,0.27)	1	0.63 (0.57,0.69)	1
Respondent edu. Level	0.15 (-0.1,0.42)	0.88	0.19 (-0.05,0.44)	0.943	0.38 (0.24,0.52)	1	-0.12 (-0.28,0.04)	0.063	0.34 (0.18,0.5)	1
Wealth Index†	0.1 (-0.06,0.26)	0.881	-0.08 (-0.21,0.05)	0.105	-0.03 (-0.13,0.07)	0.302	0.06 (-0.02,0.14)	0.92	0.05 (-0.08,0.18)	0.783
**Level 2a (Interviewer)**										
Interviewer age‡	0.05 (0.01,0.09)	0.993	0 (-0.08,0.07)	0.455	-0.03 (-0.11,0.05)	0.226	-0.02 (-0.04,0.01)	0.095	0 (-0.05,0.06)	0.571
Interviewer survey experience	-0.18 (-0.79,0.44)	0.276	0.24 (-1.36,1.79)	0.624	0.02 (-1.34,1.38)	0.514	-0.52 (-1.01,-0.03)	0.02	0.1 (-0.63,0.82)	0.607
**Level 2b (Survey cluster)**										
Rural	-0.39 (-0.8,0.03)	0.033	0.29 (-0.08,0.66)	0.94	0.18 (-0.06,0.41)	0.93	-0.4 (-0.64,-0.17)	< 0.001	0.13 (-0.24,0.49)	0.754
**Variance Components**										
Cluster-specific variance	0.128 (0, 0.599)		0.416 (0.099, 0.891)		0.316 (0.095, 0.601)		0.022 (0, 0.13)		0.274 (0.066, 0.566)	
Interviewer-specific variance	0.627 (0.256, 1.316)		0.414 (0.12, 1.134)		4.575 (3.113, 6.74)		0.176 (0.066, 0.432)		0.671 (0.385, 1.132)	
# of clusters	415		362		1283		485		546	
# of fieldworkers	50		19		74		25		50	
# of observations	1693		1341		5105		2857		2906	

* Respondent age in years centered at 15
† Wealth index centered at 2 of 5, with 5 being highest relative wealth
‡ Interviewer age in years centered at 18

Table S10. Log odds, 95% credible intervals, and posterior probability that log odds are greater than 0 from sensitivity model of ever reporting sexual intercourse among never-unioned men, using age difference of five or more years (Continued)

	**Timor-Leste (2016)**		**Uganda (2016)**		**South Africa (2016)**		**Zambia (2018)**		**Zimbabwe (2015)**	
	Estimate (95% CrI)	Posterior Prob. > 0	Estimate (95% CrI)	Posterior Prob. > 0	Estimate (95% CrI)	Posterior Prob. > 0	Estimate (95% CrI)	Posterior Prob. > 0	Estimate (95% CrI)	Posterior Prob. > 0
Intercept	-2.74 (-4.44,-1.11)		-1.71 (-2.57,-0.86)		-1.64 (-2.67,-0.64)		-3.06 (-5.14,-0.89)		-0.83 (-1.5,-0.18)	
**Level 1 (Respondent)**										
Age difference ≥5 years	-0.64 (-1.12,-0.16)	0.005	0.66 (0.18,1.14)	0.997	-0.48 (-1,0.03)	0.032	3.38 (2.13,4.43)	1	0.03 (-0.31,0.36)	0.566
Respondent age*	0.16 (0.13,0.2)	1	0.3 (0.25,0.35)	1	0.22 (0.18,0.27)	1	0.35 (0.32,0.38)	1	0.29 (0.26,0.33)	1
Respondent edu. Level	0.4 (0.21,0.61)	> 0.999	0.34 (0.15,0.53)	> 0.999	1.24 (0.92,1.58)	1	0.29 (0.15,0.44)	> 0.999	-0.22 (-0.4,-0.04)	0.008
Wealth Index†	0.03 (-0.12,0.17)	0.647	0.1 (0,0.2)	0.979	-0.22 (-0.36,-0.09)	< 0.001	-0.09 (-0.17,-0.01)	0.018	0.06 (-0.04,0.16)	0.871
**Level 2a (Interviewer)**										
Interviewer age‡	0.01 (-0.11,0.13)	0.597	-0.03 (-0.06,0.01)	0.078	0.02 (-0.01,0.05)	0.895	-0.03 (-0.06,0.01)	0.048	-0.01 (-0.04,0.02)	0.231
Interviewer survey experience	0.19 (-1.19,1.61)	0.614	-0.34 (-0.89,0.2)	0.102	-0.1 (-0.75,0.54)	0.373	-0.82 (-2.6,0.96)	0.176	-0.39 (-0.7,-0.09)	0.005
**Level 2b (Survey cluster)**										
Rural	-0.77 (-1.24,-0.31)	< 0.001	0.18 (-0.12,0.49)	0.884	-0.12 (-0.51,0.27)	0.275	0.43 (0.21,0.65)	> 0.999	0.05 (-0.25,0.34)	0.618
**Variance Components**										
Cluster-specific variance	0.51 (0.147, 1.073)		0.183 (0.009, 0.465)		0.321 (0.026, 0.762)		0.198 (0.09, 0.336)		0.185 (0.069, 0.338)	
Interviewer-specific variance	2.437 (1.226, 4.831)		0.31 (0.147, 0.599)		0.282 (0.095, 0.647)		0.592 (0.344, 1.018)		0.114 (0.048, 0.232)	
# of clusters	387		628		626		544		394	
# of fieldworkers	32		39		30		39		60	
# of observations	1712		2029		2241		4934		3265	

* Respondent age in years centered at 15
† Wealth index centered at 2 of 5, with 5 being highest relative wealth
‡ Interviewer age in years centered at 18

	Observations	Marginal Probability at Survey Exp. None	Any	% Difference (95% CrI)
**Cameroon (2018)**				
Women	4915	52.4%	58.5%	-6.1 (-13.9, 1.9)
Men	3380	56.5%	59.9%	-3.4 (-16.4, 10.3)
**Zimbabwe (2015)**				
Women	2386	26.6%	32.5%	-5.8 (-12.5, 1.3)
Men	3265	45.8%	53%	-7.1 (-13.1, -1.3)
**Uganda (2016)**				
Women	4567	42.7%	47%	-4.2 (-9.5, 1.2)
Men	2029	55.8%	61.7%	-6 (-16, 4.5)
**Liberia (2019-20)**				
Women	2621	80.3%	84.3%	-4.1 (-8.2, 0.1)
Men	1543	65.1%	65.7%	-0.6 (-9.9, 8.9)
**Burundi (2016-17)**				
Women	6091	16.4%	19.9%	-3.2 (-10.7, 3.6)
Men	2825	27.8%	28%	1.9 (-28.7, 24.1)
**Sierra Leone (2019)**				
Women	4966	67.9%	70.4%	-2.6 (-8.5, 3.4)
Men	2906	63%	62.1%	0.9 (-7.6, 9.5)
**Timor-Leste (2016)**				
Women	4412	3.2%	4.5%	-1.2 (-3.2, 0.9)
Men	1712	28.3%	26.8%	2 (-13.2, 16.9)
**Philippines (2017)**				
Women	8652	14.9%	15.8%	-0.8 (-4.7, 3.1)
**Zambia (2018)**				
Women	4105	58.7%	59.3%	-0.6 (-6.7, 5.5)
Men	4934	64.7%	75.3%	-12 (-31, 12.5)
**Rwanda (2019-20)**				
Women	5961	33%	33.6%	-0.6 (-3.4, 2.3)
Men	2857	36.4%	46.3%	-9.8 (-19, -0.8)
**Nigeria (2018)**				
Women	10669	33.1%	33.5%	-0.1 (-11.7, 11.4)
Men	5105	32.6%	32.3%	0.6 (-13.7, 14.1)
**Gambia (2019-20)**				
Women	3226	12.3%	12.2%	0.2 (-3, 3.3)
Men	2384	46.7%	44.6%	2.1 (-7.8, 12)
**Ethiopia (2016)**				
Women	4250	13.2%	12.9%	0.4 (-3, 3.7)
Men	4600	28.1%	36.1%	-7.7 (-22, 5.2)
**South Africa (2016)**				
Women	5134	80.2%	78.8%	1.4 (-2.4, 5.5)
Men	2241	80%	79.2%	0.6 (-7, 9)
**Guinea (2018)**				
Women	2645	33.9%	32.7%	1.4 (-9.2, 11.3)
Men	1761	46.6%	52.4%	-5.7 (-25.1, 15.1)
**Malawi (2015-16)**				
Women	4993	44.6%	42.9%	1.7 (-3.7, 6.8)
Men	2666	62.5%	63.9%	-1.5 (-8.8, 6)
**Benin (2017-18)**				
Women	3897	49.3%	47.4%	1.9 (-4.7, 8.5)
Men	2906	53%	56.8%	-3.7 (-14, 6.6)
**Haiti (2017-18)**				
Women	5760	56.5%	53.1%	3.4 (-2.6, 9.5)
Men	4608	75.6%	74.3%	1.2 (-3.5, 6.3)
**Mali (2018)**				
Women	1816	35.8%	31.9%	4.2 (-6.9, 14.9)
Men	1580	43.3%	37.5%	6 (-10.3, 21.9)
**Nepal (2016)**				
Men	1341	30%	27%	4.4 (-19.4, 23.4)
**Myanmar (2015-16)**				
Men	1693	11.5%	14%	-2.2 (-8, 3.5)

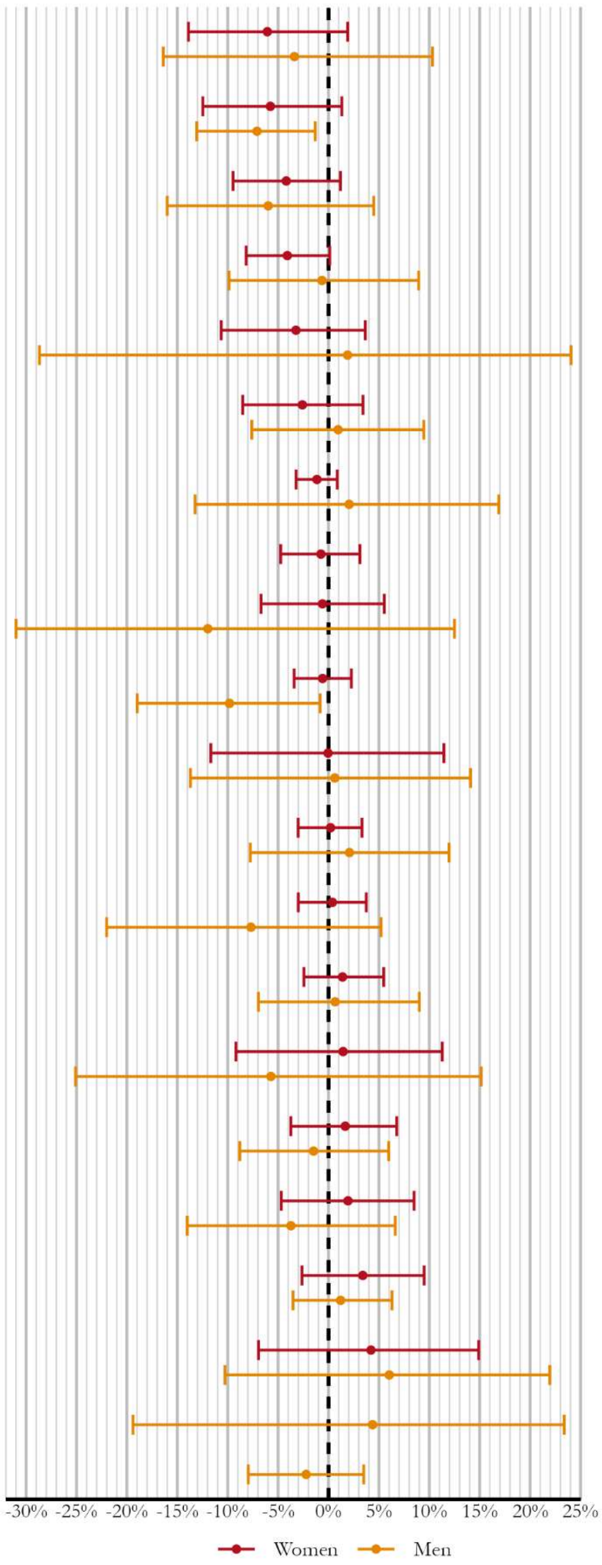

Figure S1 Average Marginal Effect contrasts of reporting ever having sexual intercourse among never-union respondents when an interviewer has previous survey experience and does not have survey experience, adjusting for respondent and interviewer characteristics.
